# Supplementary material for: Chemical shift prediction of RNA imino groups: application toward characterizing RNA excited states
Source: Nat Commun. 2021 Mar 11;12:1595. doi: 10.1038/s41467-021-21840-x (PMC7952389; doi:10.1038/s41467-021-21840-x)
Supplement: Supplementary file 1 — Supplementary Information [file 41467_2021_21840_MOESM1_ESM.pdf]

Supplementary Information

Chemical Shift Prediction of RNA Imino Groups: Application toward Characterizing RNA Excited States

Yanjiao Wang,[1] Ge Han,[1] Xiuying Jiang,[1] Tairan Yuwen,[2] Yi Xue*[1]

[1] School of Life Sciences; Tsinghua-Peking Joint Center for Life Sciences; Beijing Advanced Innovation Center for Structural Biology, Tsinghua University, Beijing, 100084, China

[2] Department of Pharmaceutical Analysis & State Key Laboratory of Natural and Biomimetic Drugs, School of Pharmaceutical Sciences, Peking University, Beijing 100191, China

Table of Contents

TABLE OF CONTENTS **1**

SUPPLEMENTARY FIGURES **2**

FIGURE S1 2
FIGURE S2 3
FIGURE S3 4
FIGURE S4 5
FIGURE S5 6
FIGURE S6 7
FIGURE S7 8
FIGURE S8 9
FIGURE S9 39
FIGURE S10 40
FIGURE S11 41
FIGURE S12 42
FIGURE S13 43
FIGURE S14 44
FIGURE S15 45

SUPPLEMENTARY TABLES **46**

TABLE S1 46
TABLE S2 47
TABLE S3 49
TABLE S4 50
TABLE S5 50
TABLE S6 51
TABLE S7 52
TABLE S8 53
TABLE S9 53

Supplementary Figures

Figure S1. The $^{1}H^{N}$-^{15}N resonance distributions of BP-triplets with the most occurrences in the training dataset. The BP-triplet name is shown in the top-left corner of each box. The left number in the bracket indicates the total occurrence of the specific BP-triplet, and the right number is the same except that the BP-triplets flanked by the same base pairs are counted only once. In each box, most resonances cluster very well around an average position, while those discrete ones are outliers that will be trimmed according to the three-sigma rule.

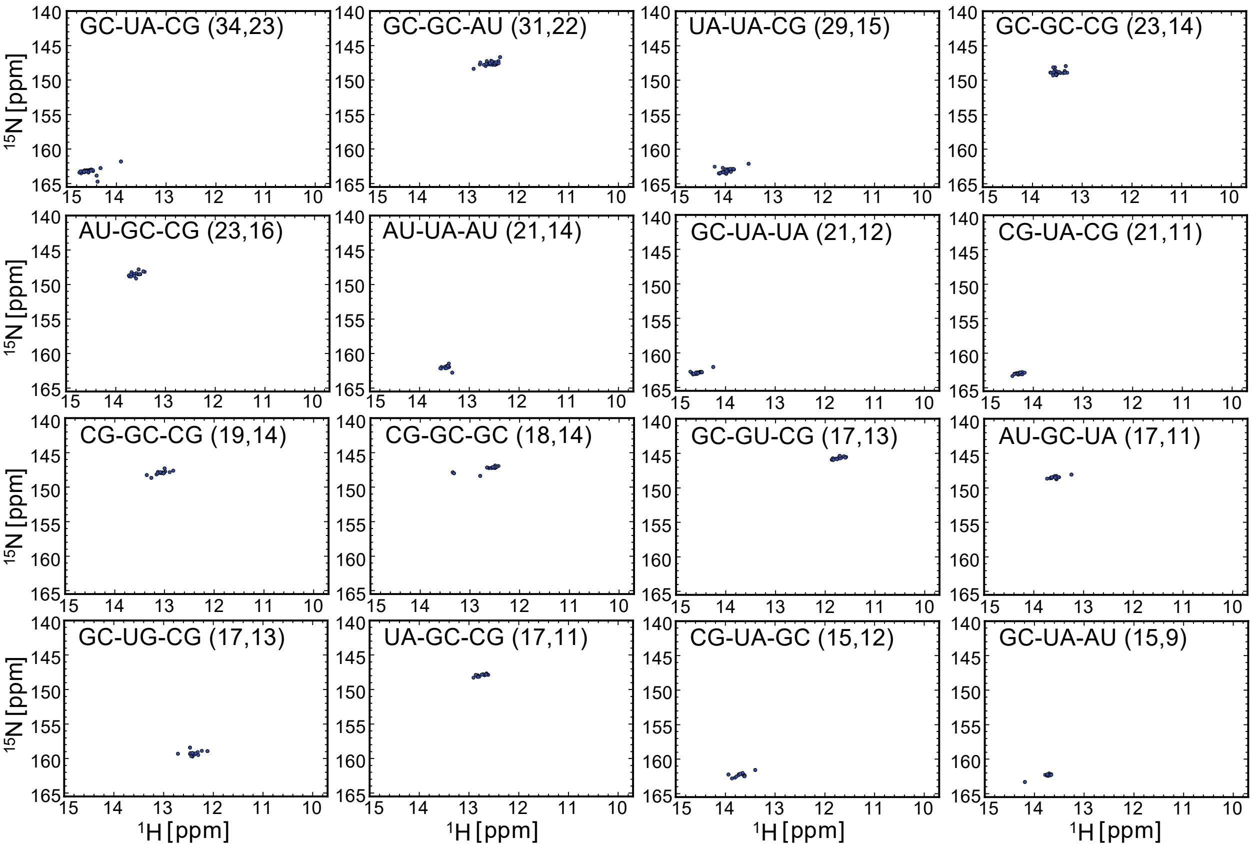

Figure S2. Correlation between LARMOR^D predicted and experimental chemical shifts for ^{15}N (left) and $^1H^N$ (right) in the training dataset (a) and the testing dataset (b).

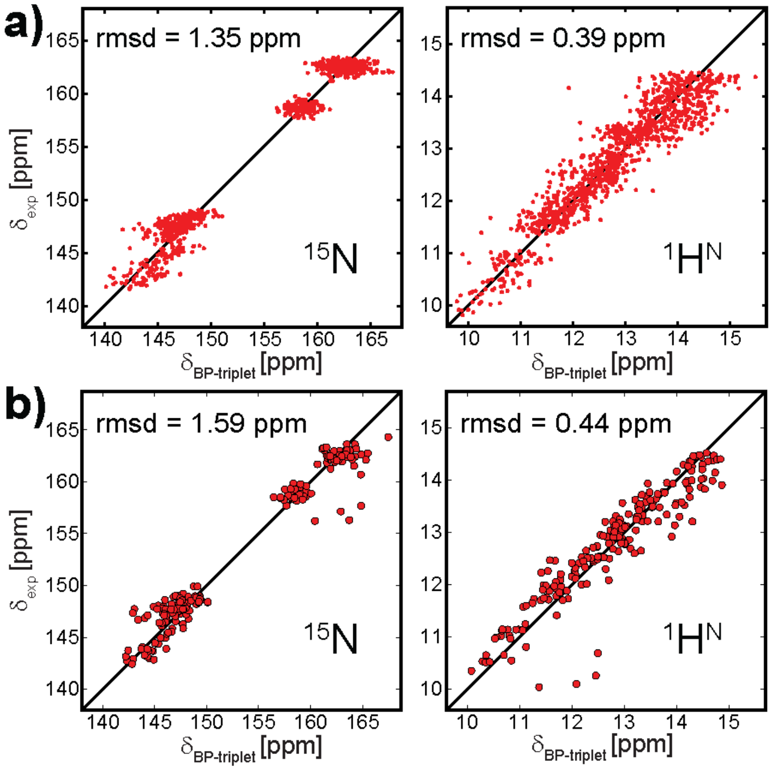

Figure S3. Correlation between chemical shifts measured at 10 °C and 25 °C. The chemical shift data were collected by measuring ^{1}H-^{15}N 2D spectra at 10 °C and 25 °C for nine hairpin samples (HP17, HP19, HP20, HP22, HP23, HP24, HP25, HP26 and HP28). The offset is shown by a dashed line along the data points. After re-referencing, the rmsd values for ^{15}N and $^{1}H^{N}$ can be reduced to 0.079 ppm and 0.029 ppm, respectively.

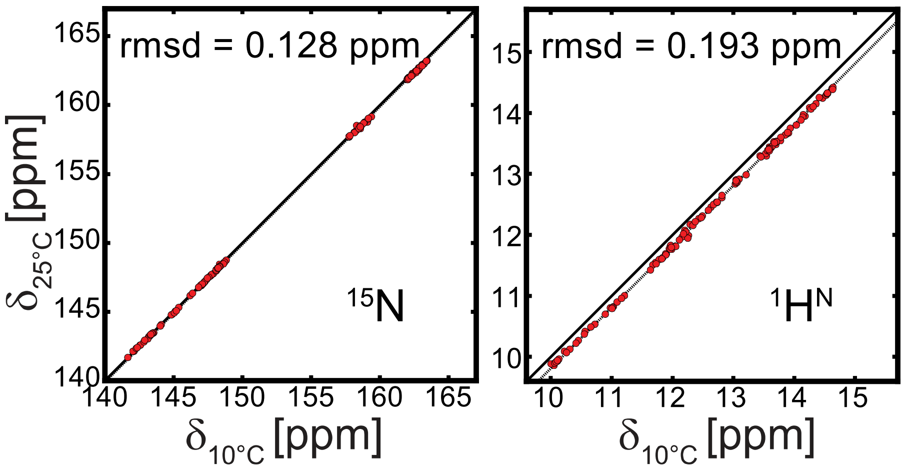

Figure S4. Enlarged imino chemical shift maps of BP-triplets (i.e., Fig. 2). The chemical shift distributions of BP-triplets with different central base pairs are displayed in four subplots, respectively. In each subplot, the average imino resonance of each BP-triplet is marked on the map. The error bars represent one standard deviation (s.d.) (the corresponding sample sizes are provided in Supplementary Table 2). Each BP-triplet is described as a triplet code: the middle code denotes the central base pair of interest; the first code and the last code are 5′ and 3′ neighbouring base pairs, and are represented by corresponding colors and shapes, respectively.

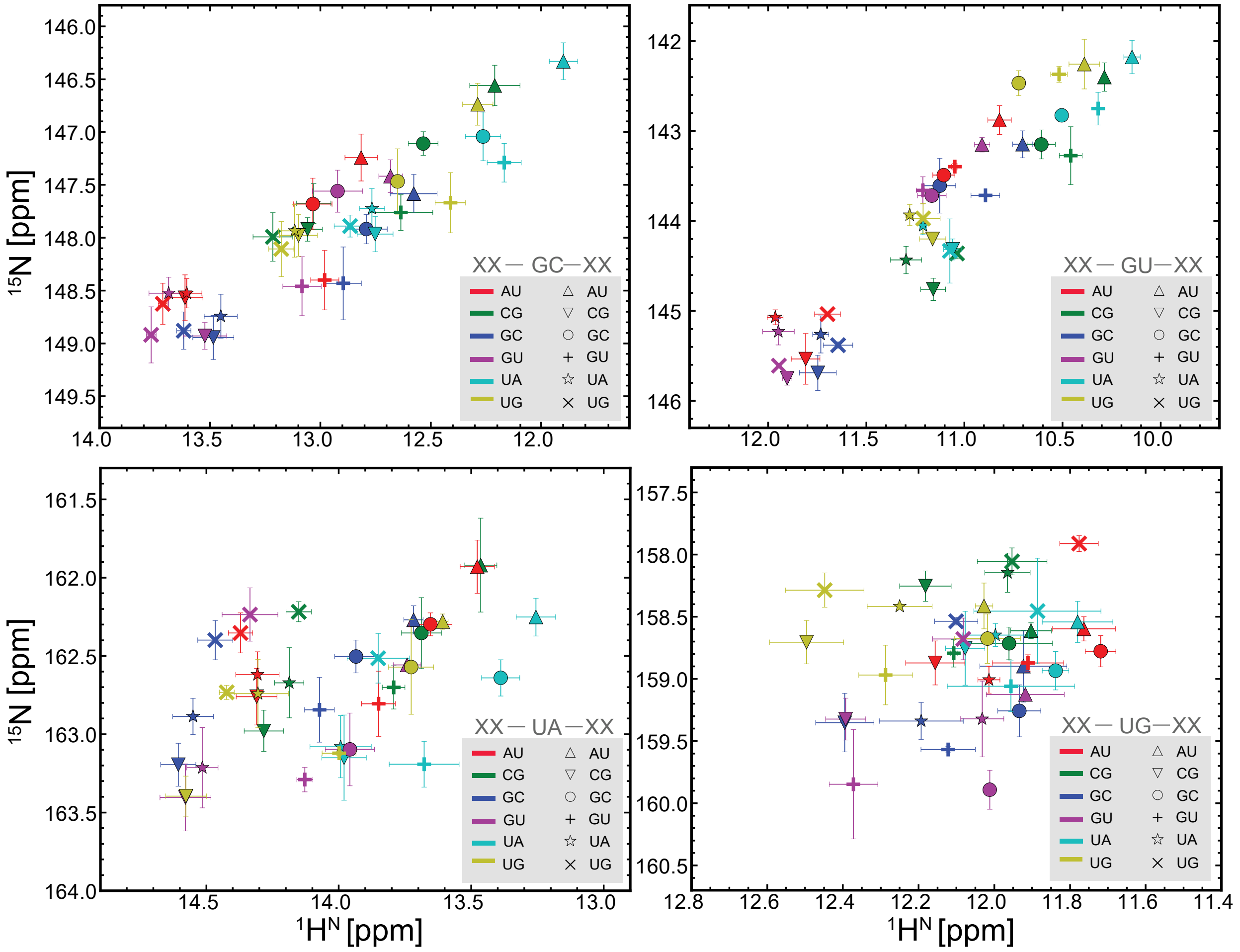

Figure S5. RC and EF shifts calculated by the semi-empirical model using structures built by RNAComposer. The DC parameter set (see the main text) is used here. Chemical shift data of various BP-triplets are divided into four groups according to the central base pairs, as shown in different colors of x-axis labels. For each BP-triplet, RC shift (dark blue) and EF shift (black) are shown side by side.

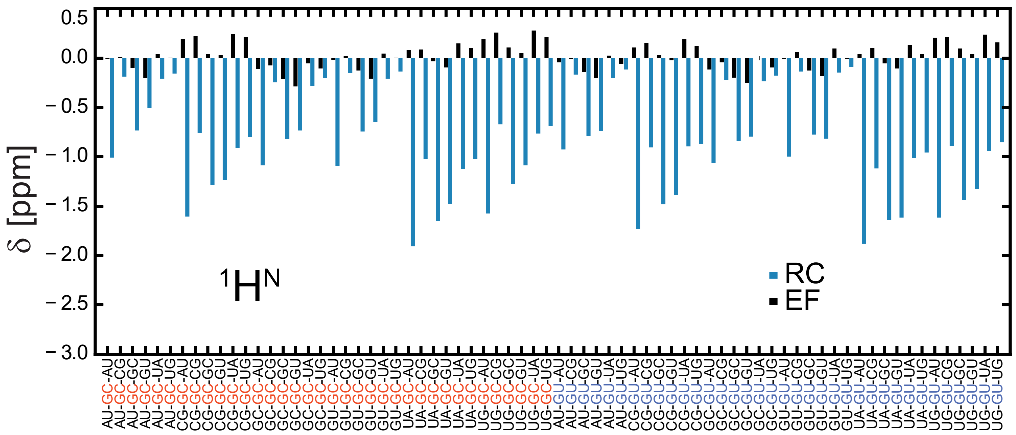

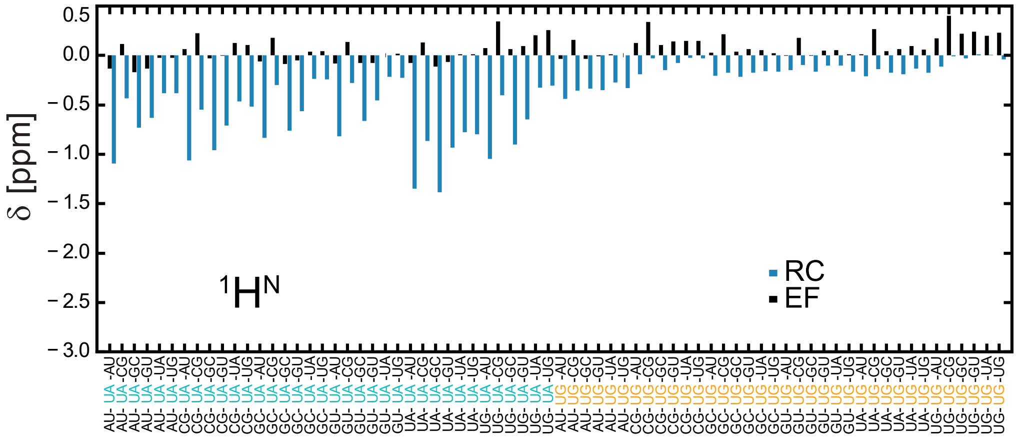

Figure S6. Correlation between chemical shifts calculated by the semi-empirical model (including both RC and EF effects) and chemical shifts in BP-triplet lookup table. The structure models were generated by RNAComposer. Chemical shift data in BP-triplet table are divided into four groups (GC: red; GU: blue; UA: cyan; UG: orange) according to the central base pair. The upper four subplots show correlation for ^{15}N, and the lower four subplots show correlation for $^{1}H^{N}$. The semi-empirical calculation was carried out by summing up the intrinsic chemical shifts of the central base pair and RC/EF contributions of two neighbouring base pairs. For each subplot, the intrinsic chemical shift was adjusted so that the semi-empirical result and data from the BP-triplet table give the same mean value.

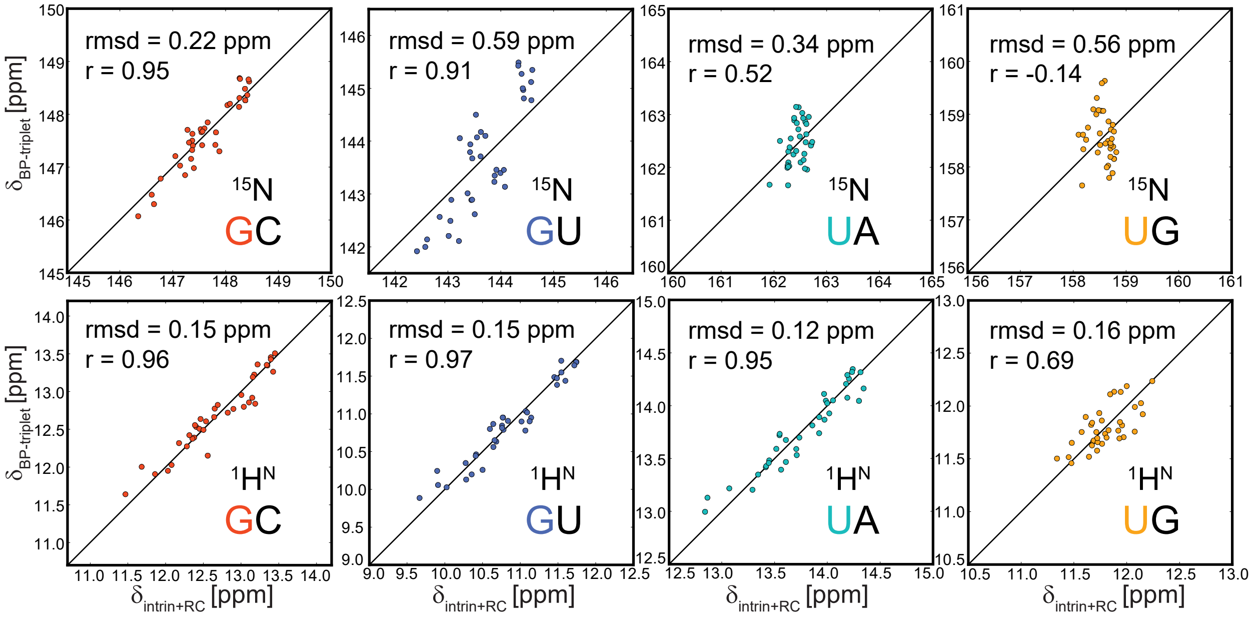

Figure S7. Comparison of chemical shifts from semi-empirical calculations and the lookup table. (a) Correlation between chemical shifts calculated by the semi-empirical model using crystal structures and chemical shifts in the BP-triplet lookup table. Note that 108 (instead of 144) different BP-triplets can be found in the crystal structures. (b) Correlation between the average chemical shifts calculated by the semi-empirical model using crystal structures and chemical shifts in the BP-triplet lookup table. The only difference from panel ‘a’ is that the chemical shifts from the same BP-triplet are averaged. (c) Correlation between chemical shifts calculated by the semi-empirical model using structures built by 3DNA and chemical shifts in BP-triplet lookup table. 3DNA structures contain $4 \times 4 \times 4 = 64$ Watson-Crick BP-triplets. (d) Correlation between chemical shifts calculated by the semi-empirical model using structures built by RNAComposer and chemical shifts in BP-triplet lookup table (same as Fig. 3a, but only 64 WC BP-triplets are presented to make a fair comparison with 3DNA result).

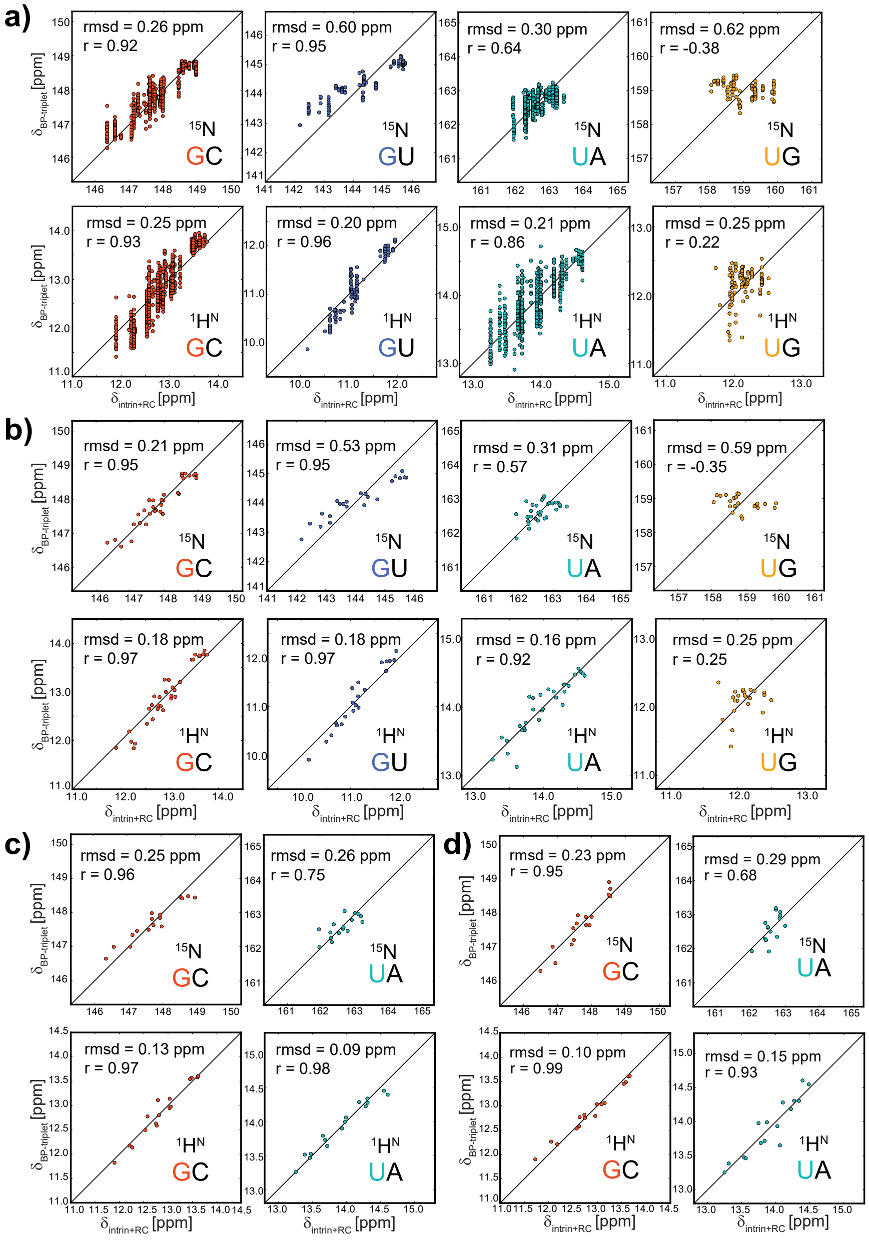

Figure S8. Rigid-body parameters for BP-triplets in crystal, RNAComposer and 3DNA structures.

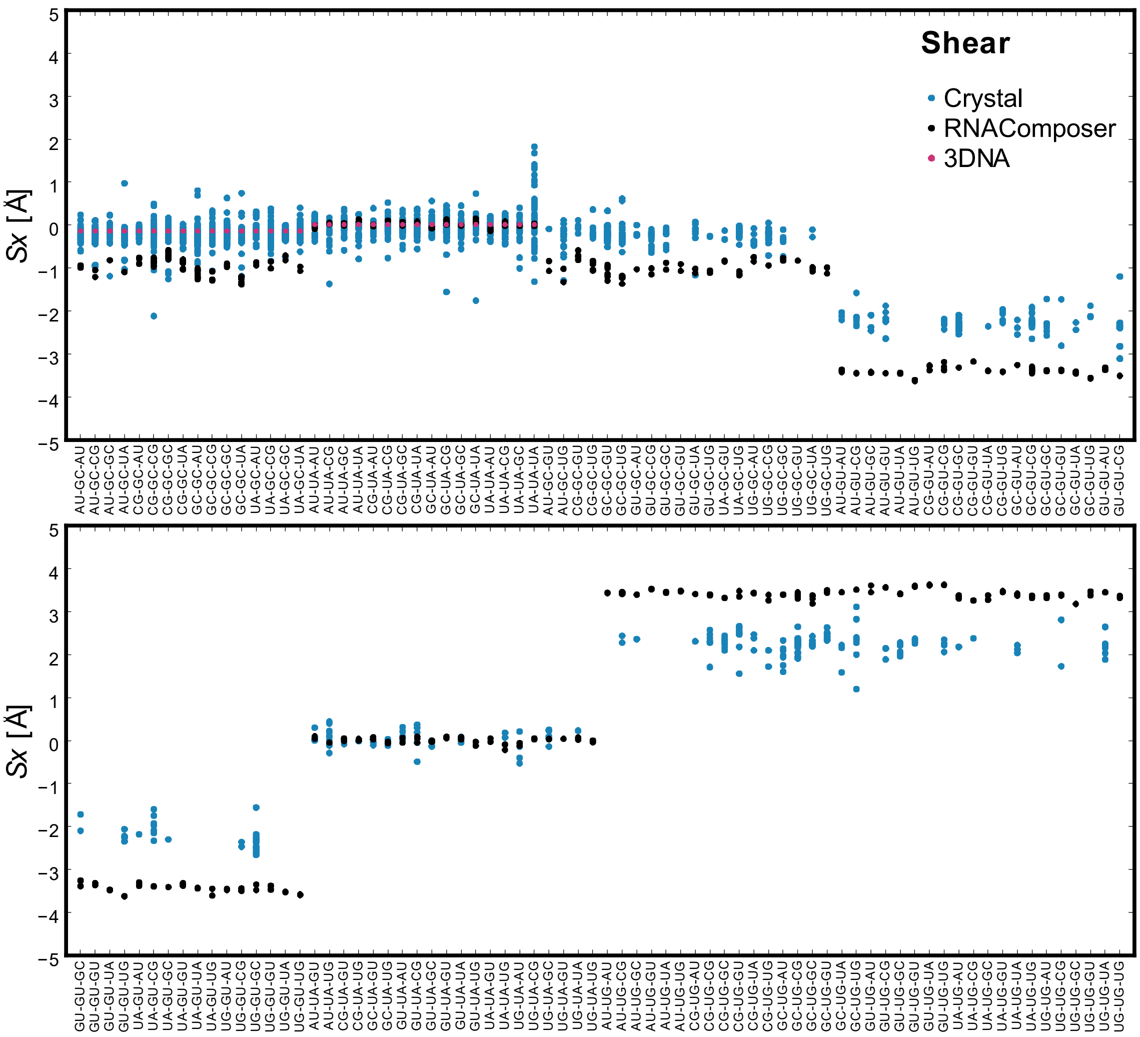

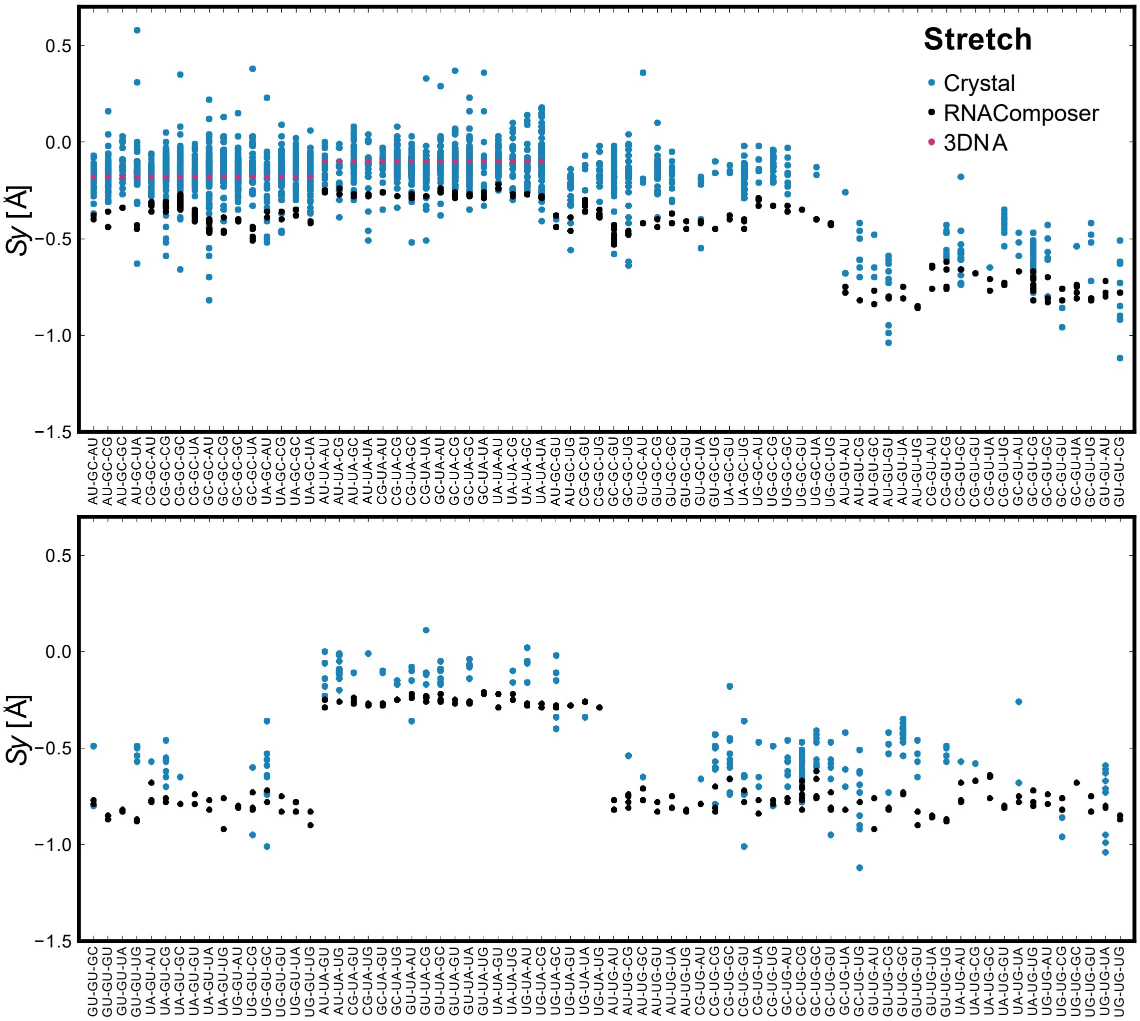
Stretch
Crystal
RNAComposer
3DNA
Sy [Å]
0.5
0.0
−0.5
−1.0
−1.5
Sy [Å]
0.5
0.0
−0.5
−1.0
−1.5

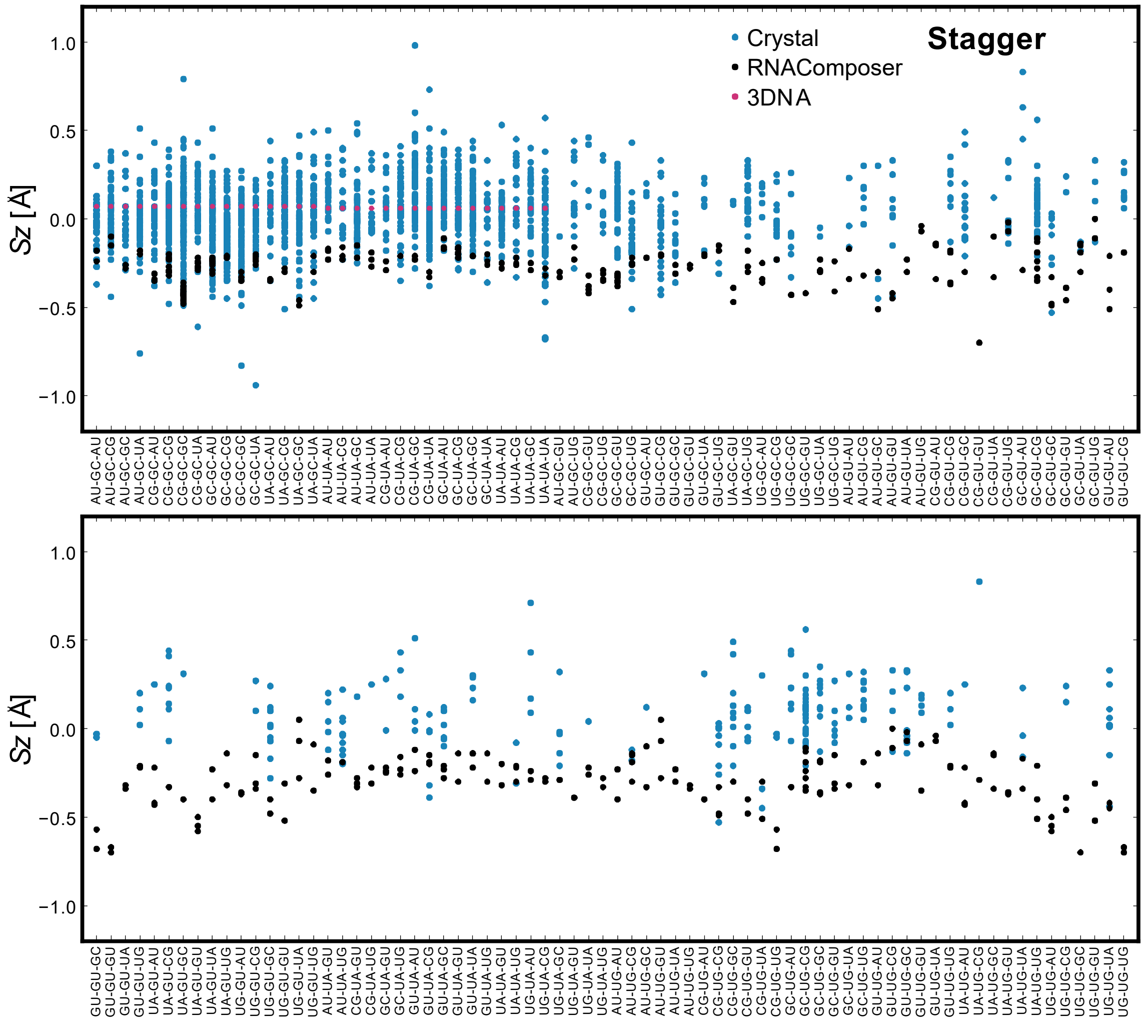

Crystal
RNAComposer
3DNA
Stagger
Sz [Å]
1.0
0.5
0.0
−0.5
−1.0

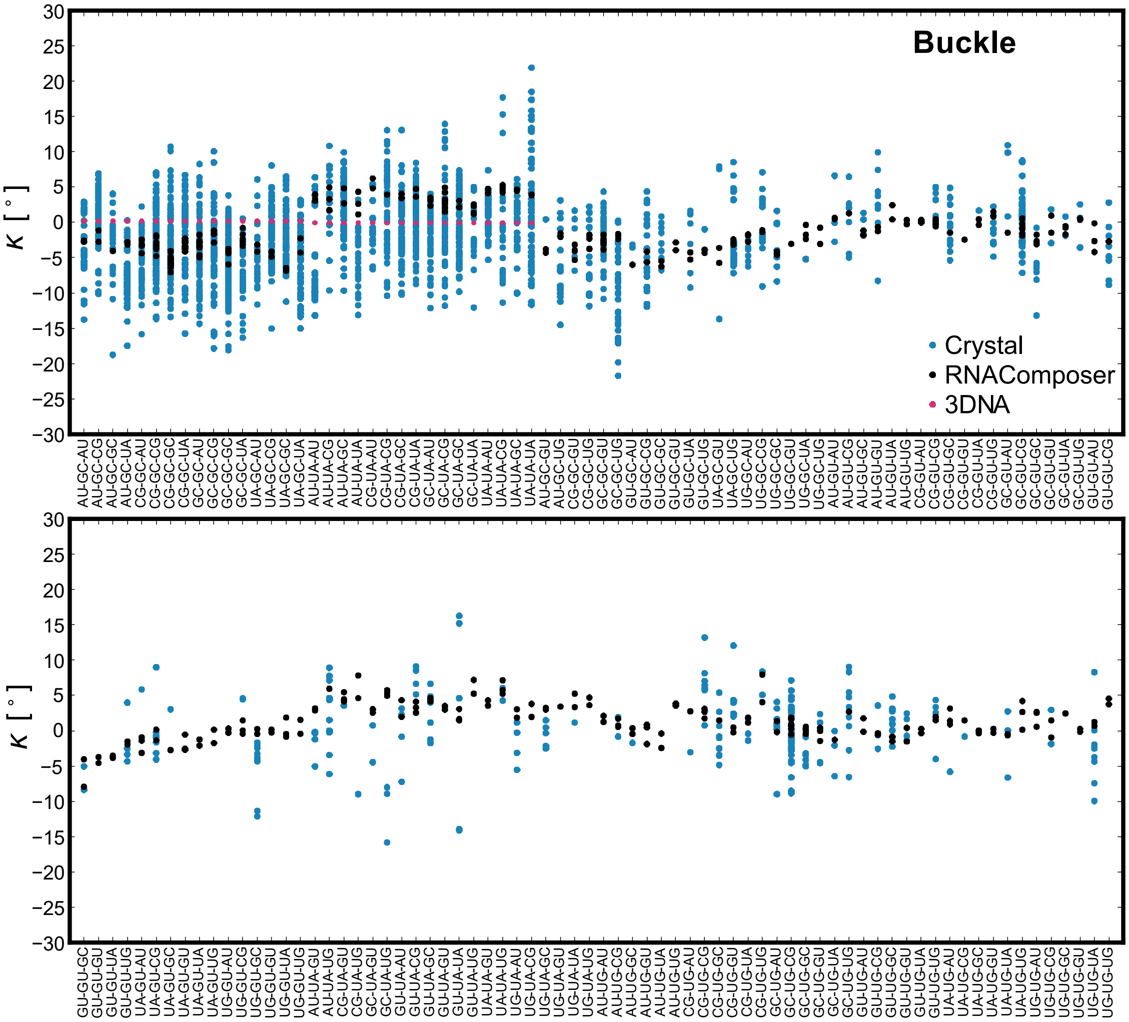
Buckle
Crystal
RNAComposer
3DNA
κ [°]
κ [°]

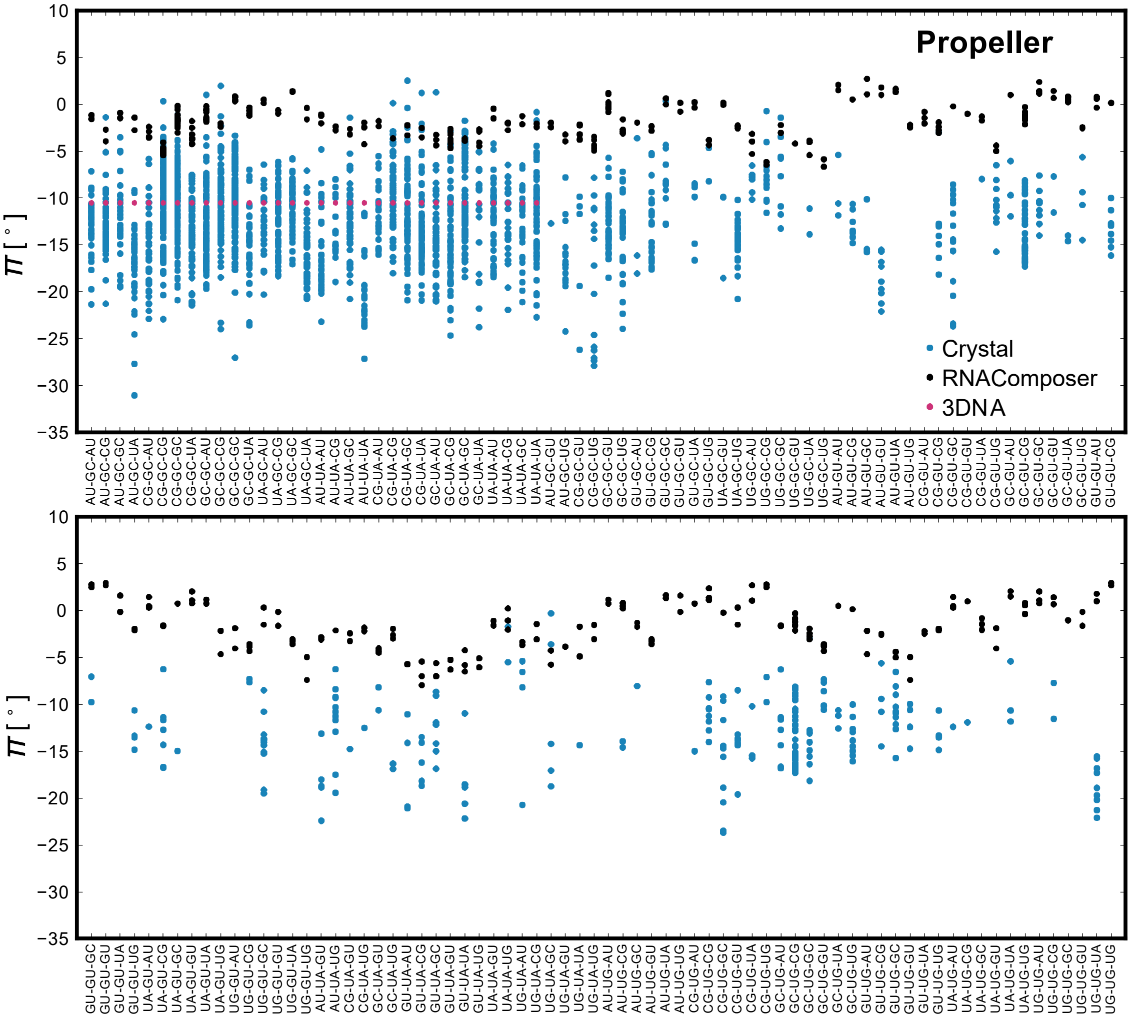

Propeller
Crystal
RNAComposer
3DNA

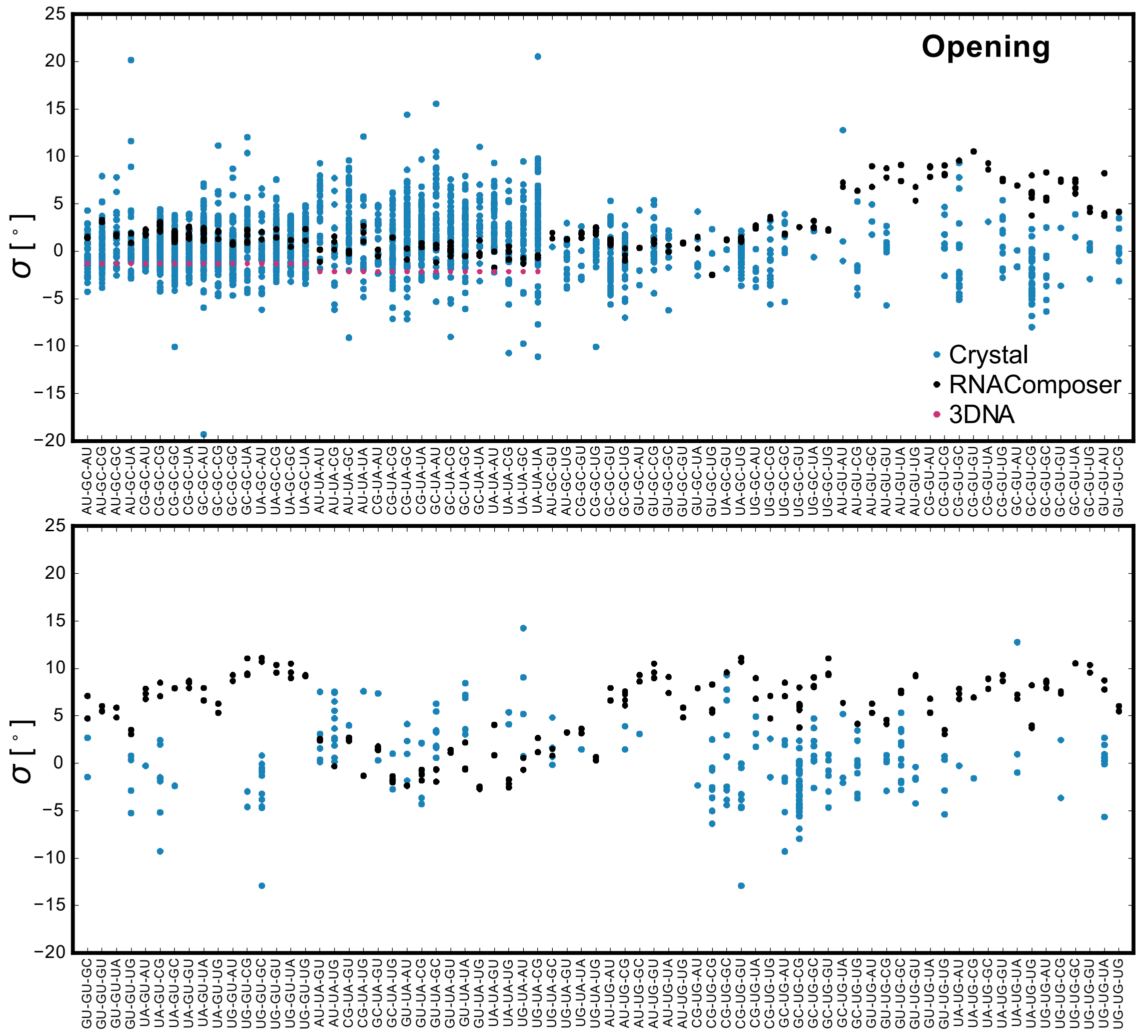
Opening
Crystal
RNAComposer
3DNA
σ [°]

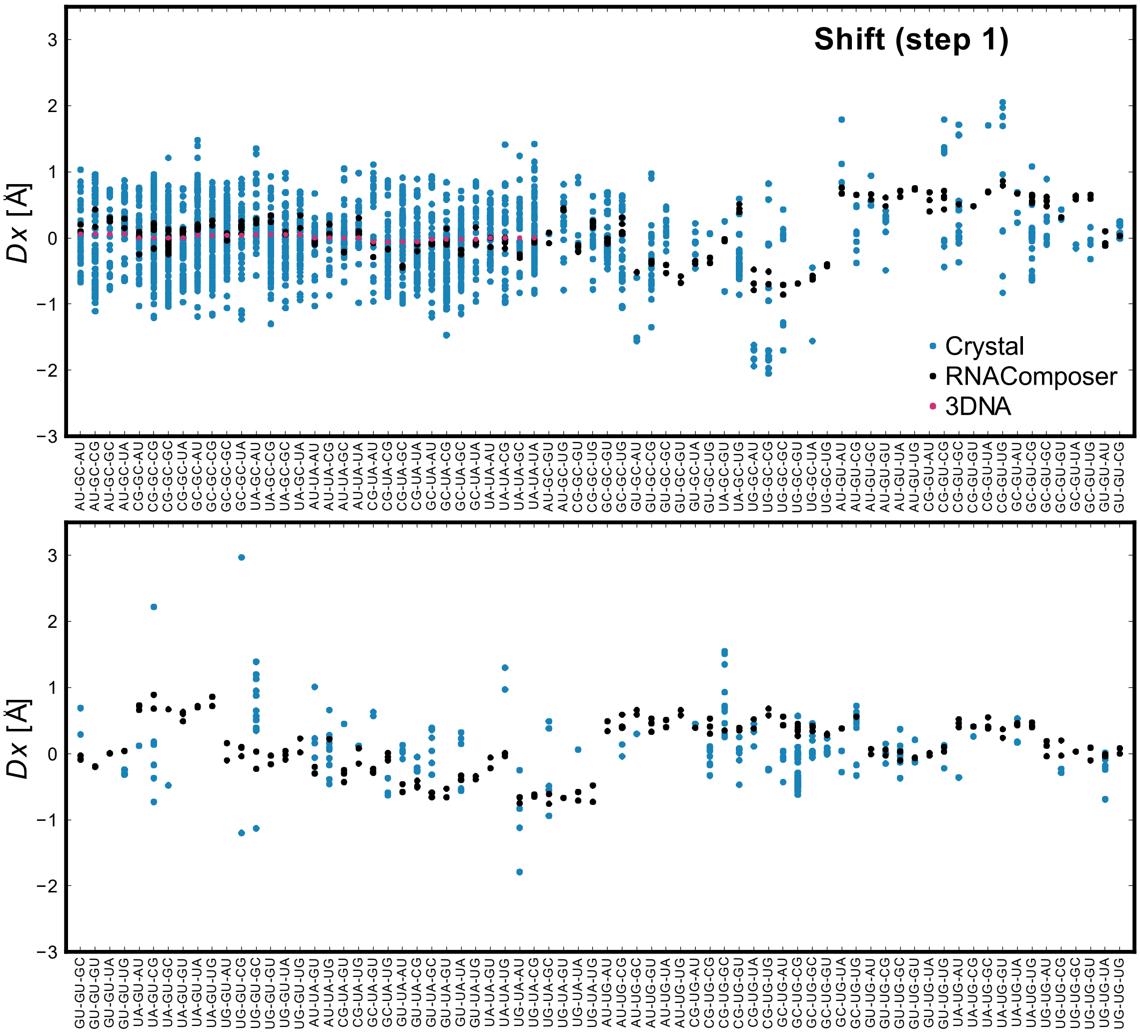
Shift (step 1)
Dx [Å]
3
2
1
0
−1
−2
−3
Crystal
RNAComposer
3DNA
AU-GC-AU AU-GC-CG AU-GC-GC AU-GC-UA CG-GC-AU CG-GC-CG CG-GC-GC CG-GC-UA GC-GC-AU GC-GC-CG GC-GC-GC GC-GC-UA UA-GC-AU UA-GC-CG UA-GC-GC UA-GC-UA AU-UA-AU AU-UA-CG AU-UA-GC AU-UA-UA CG-UA-AU CG-UA-CG CG-UA-GC CG-UA-UA GC-UA-AU GC-UA-CG GC-UA-GC GC-UA-UA UA-UA-AU UA-UA-CG UA-UA-GC UA-UA-UA AU-GC-GU AU-GC-UG CG-GC-GU CG-GC-UG GC-GC-GU GC-GC-UG GU-GC-AU GU-GC-CG GU-GC-GC GU-GC-GU GU-GC-UA GU-GC-UG UA-GC-GU UA-GC-UG UG-GC-AU UG-GC-CG UG-GC-GC UG-GC-GU UG-GC-UA UG-GC-UG AU-GU-AU AU-GU-CG AU-GU-GC AU-GU-GU AU-GU-UA AU-GU-UG CG-GU-AU CG-GU-CG CG-GU-GC CG-GU-GU CG-GU-UA CG-GU-UG GC-GU-AU GC-GU-CG GC-GU-GC GC-GU-GU GC-GU-UA GC-GU-UG GU-GU-AU GU-GU-CG
Dx [Å]
3
2
1
0
−1
−2
−3
GU-GU-GC GU-GU-GU GU-GU-UA GU-GU-UG UA-GU-AU UA-GU-CG UA-GU-GC UA-GU-GU UA-GU-UA UA-GU-UG UG-GU-AU UG-GU-CG UG-GU-GC UG-GU-GU UG-GU-UA UG-GU-UG AU-UA-GU AU-UA-UG CG-UA-GU CG-UA-UG GC-UA-GU GC-UA-UG GU-UA-AU GU-UA-CG GU-UA-GC GU-UA-GU GU-UA-UA GU-UA-UG UA-UA-GU UA-UA-UG UG-UA-AU UG-UA-CG UG-UA-GC UG-UA-GU UG-UA-UA UG-UA-UG AU-UG-AU AU-UG-CG AU-UG-GC AU-UG-GU AU-UG-UA AU-UG-UG CG-UG-AU CG-UG-CG CG-UG-GC CG-UG-GU CG-UG-UA CG-UG-UG GC-UG-AU GC-UG-CG GC-UG-GC GC-UG-GU GC-UG-UA GC-UG-UG GU-UG-AU GU-UG-CG GU-UG-GC GU-UG-GU GU-UG-UA GU-UG-UG UA-UG-AU UA-UG-CG UA-UG-GC UA-UG-GU UA-UG-UA UA-UG-UG UG-UG-AU UG-UG-CG UG-UG-GC UG-UG-GU UG-UG-UA UG-UG-UG

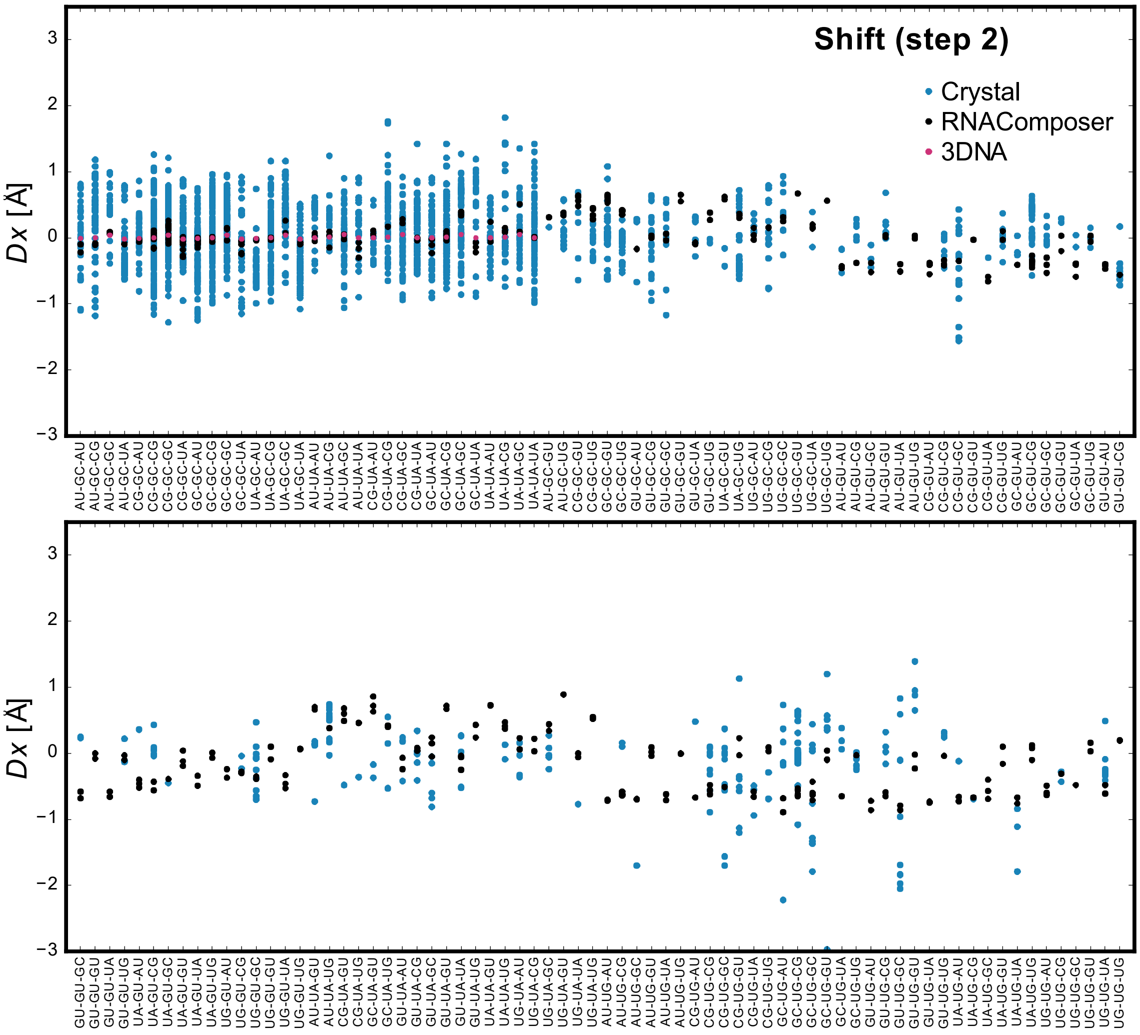
Shift (step 2)
Crystal
RNAComposer
3DNA
Dx [Å]
Dx [Å]

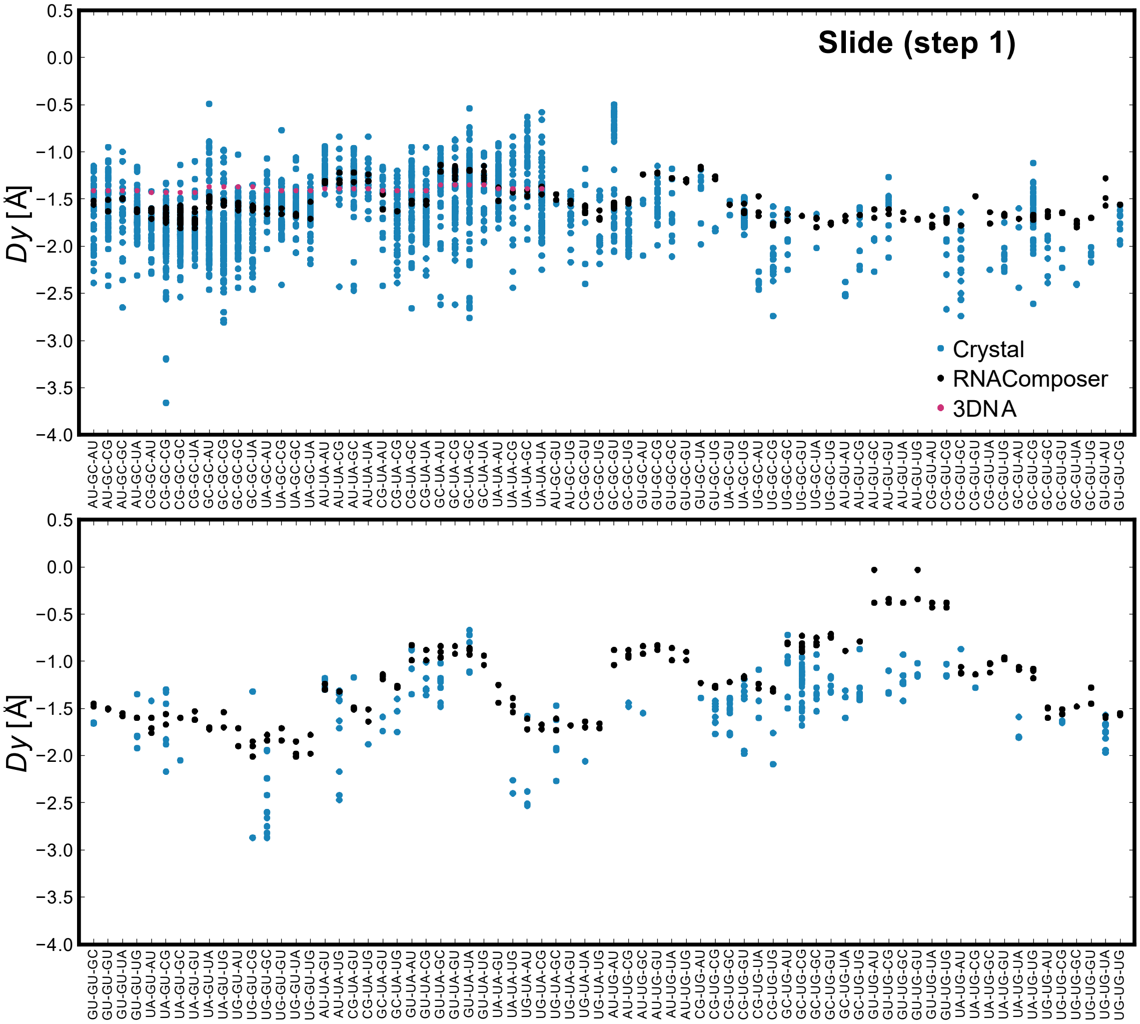

Slide (step 1)
Crystal
RNAComposer
3DNA
Dy [Å]
Dy [Å]

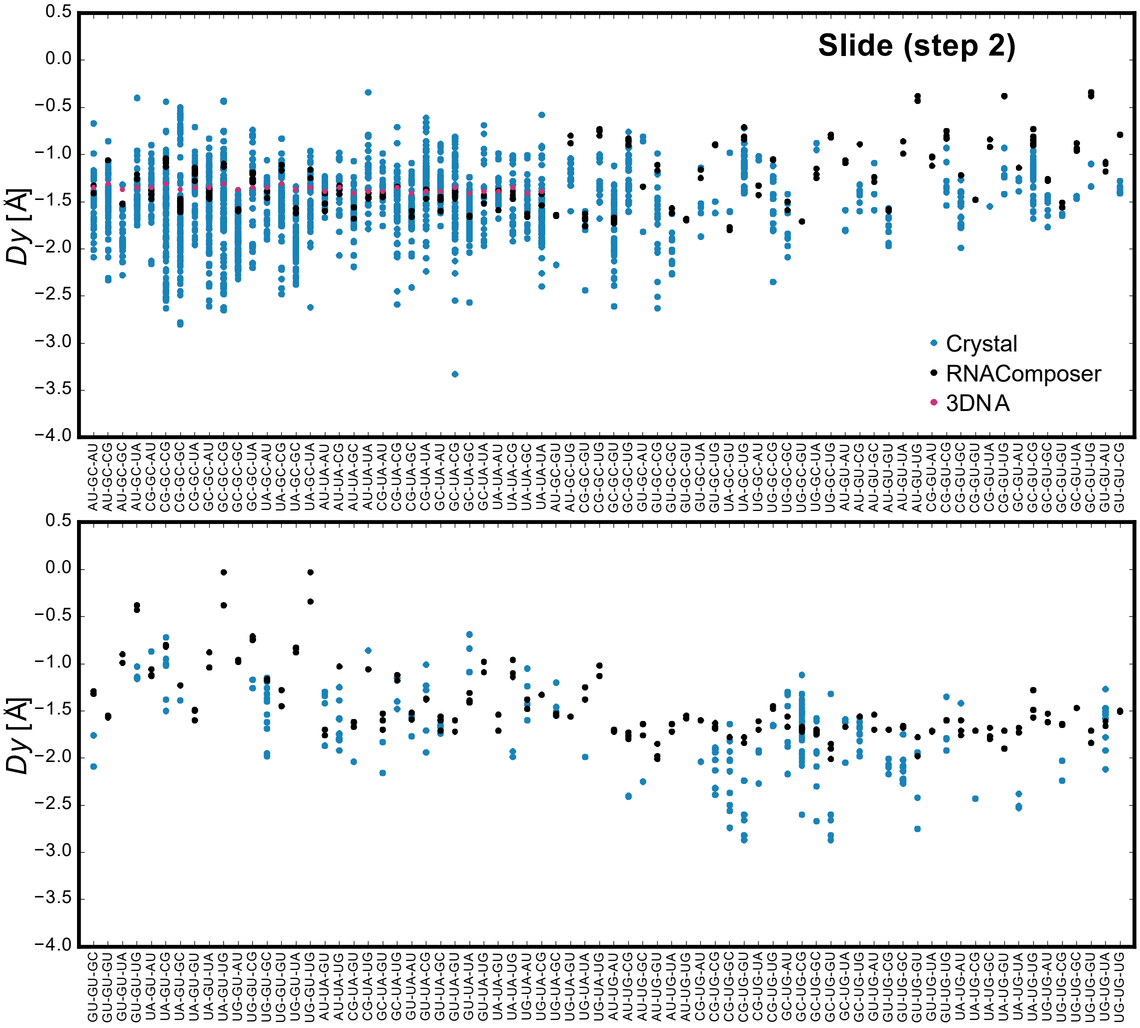
Slide (step 2)
Crystal
RNAComposer
3DNA
Dy [Å]
0.5
0.0
−0.5
−1.0
−1.5
−2.0
−2.5
−3.0
−3.5
−4.0
AU-GC-AU AU-GC-CG AU-GC-GC AU-GC-UA CG-GC-AU CG-GC-CG CG-GC-GC CG-GC-UA GC-GC-AU GC-GC-CG GC-GC-GC GC-GC-UA UA-GC-AU UA-GC-CG UA-GC-GC UA-GC-UA AU-UA-AU AU-UA-CG AU-UA-GC AU-UA-UA CG-UA-AU CG-UA-CG CG-UA-GC CG-UA-UA GC-UA-AU GC-UA-CG GC-UA-GC GC-UA-UA UA-UA-AU UA-UA-GC UA-UA-UA AU-GC-GU AU-GC-UG CG-GC-GU CG-GC-UG GC-GC-GU GC-GC-UG GU-GC-AU GU-GC-CG GU-GC-GC GU-GC-GU GU-GC-UA GU-GC-UG UA-GC-GU UA-GC-UG UG-GC-AU UG-GC-CG UG-GC-GC UG-GC-GU UG-GC-UA UG-GC-UG AU-GU-AU AU-GU-CG AU-GU-GC AU-GU-GU AU-GU-UA AU-GU-UG CG-GU-AU CG-GU-CG CG-GU-GC CG-GU-GU CG-GU-UA CG-GU-UG GC-GU-AU GC-GU-CG GC-GU-GC GC-GU-GU GC-GU-UA GC-GU-UG GU-GU-AU GU-GU-CG
GU-GU-GC GU-GU-GU GU-GU-UA GU-GU-UG UA-GU-AU UA-GU-CG UA-GU-GC UA-GU-GU UA-GU-UA UA-GU-UG UG-GU-AU UG-GU-CG UG-GU-GC UG-GU-GU UG-GU-UA UG-GU-UG AU-UA-GU AU-UA-UG CG-UA-GU CG-UA-UG GC-UA-GU GC-UA-UG GU-UA-AU GU-UA-CG GU-UA-GC GU-UA-GU GU-UA-UA GU-UA-UG UA-UA-GU UA-UA-UG UG-UA-AU UG-UA-CG UG-UA-GC UG-UA-GU UG-UA-UA UG-UA-UG AU-UG-AU AU-UG-CG AU-UG-GC AU-UG-GU AU-UG-UA AU-UG-UG CG-UG-AU CG-UG-CG CG-UG-GC CG-UG-GU CG-UG-UA CG-UG-UG GC-UG-AU GC-UG-CG GC-UG-GC GC-UG-GU GC-UG-UA GC-UG-UG GU-UG-AU GU-UG-CG GU-UG-GC GU-UG-GU GU-UG-UA GU-UG-UG UA-UG-AU UA-UG-CG UA-UG-GC UA-UG-GU UA-UG-UA UA-UG-UG UG-UG-AU UG-UG-CG UG-UG-GC UG-UG-GU UG-UG-UA UG-UG-UG

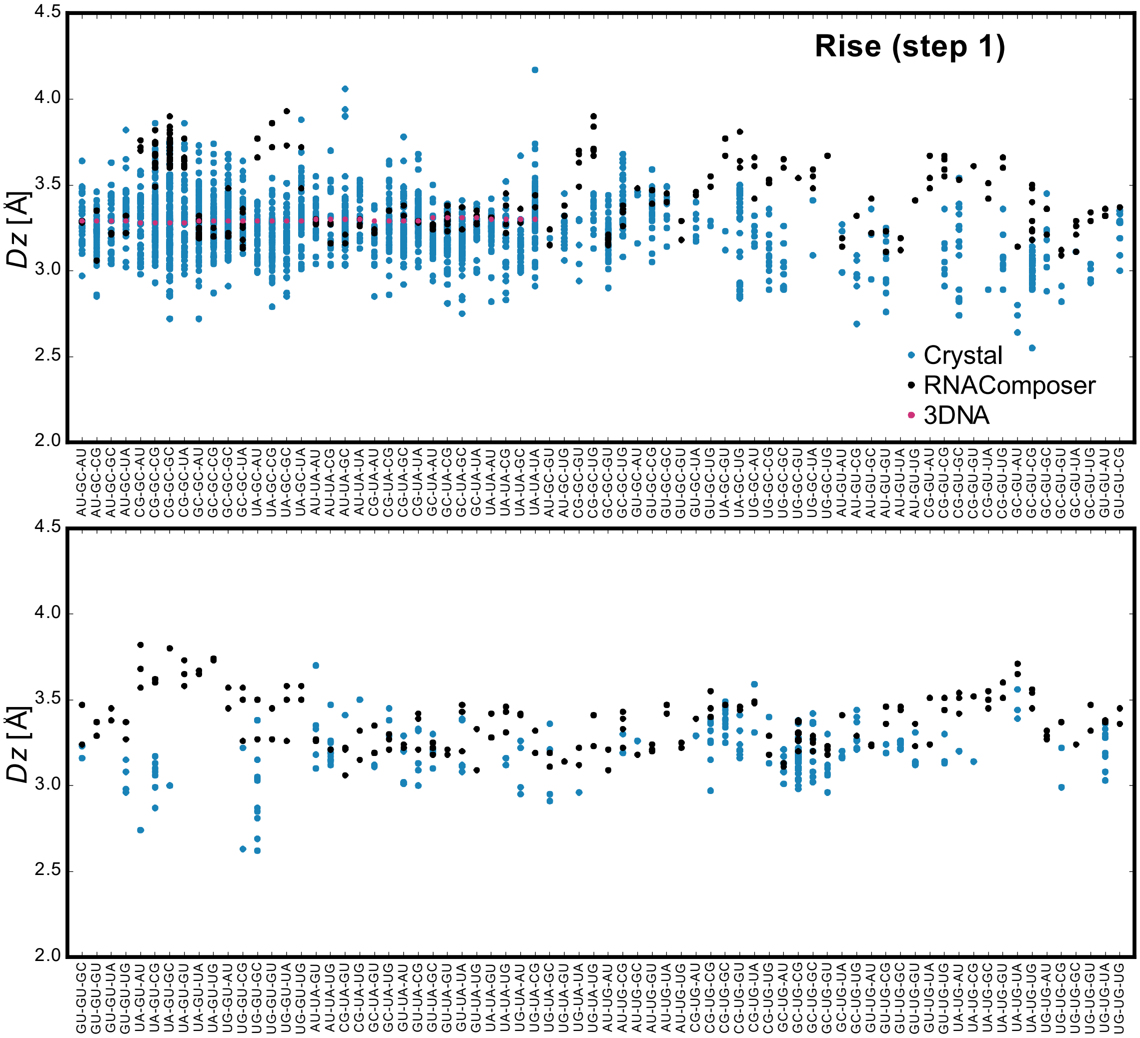

Rise (step 1)
Dz [Å]
4.5
4.0
3.5
3.0
2.5
2.0
Crystal
RNAComposer
3DNA

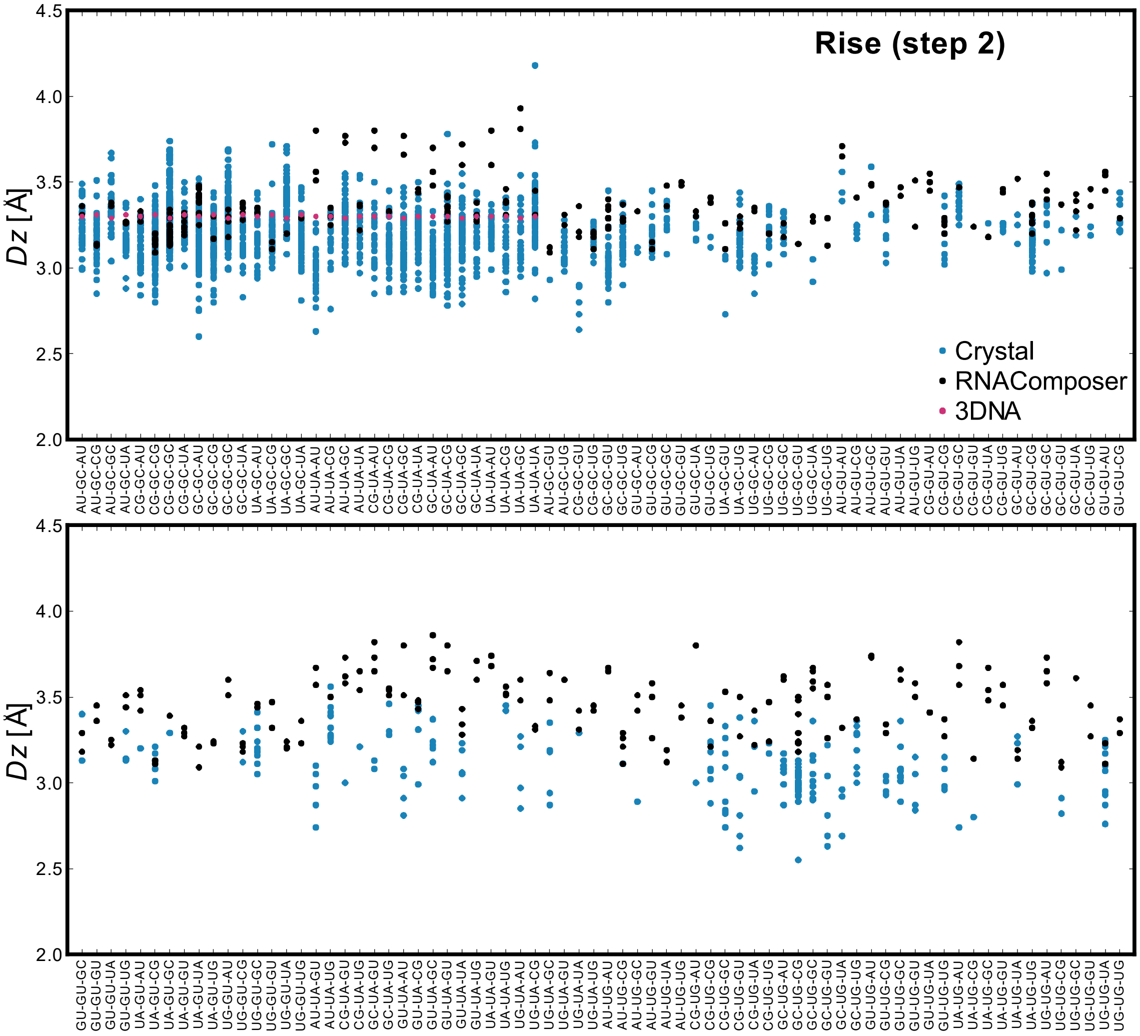

Rise (step 2)
Dz [Å]
4.5
4.0
3.5
3.0
2.5
2.0
Crystal
RNAComposer
3DNA
AU-GC-AU
AU-GC-CG
AU-GC-GC
AU-GC-UA
CG-GC-AU
CG-GC-CG
CG-GC-GC
CG-GC-UA
GC-GC-AU
GC-GC-CG
GC-GC-GC
GC-GC-UA
UA-GC-AU
UA-GC-CG
UA-GC-GC
UA-GC-UA
AU-UA-AU
AU-UA-CG
AU-UA-GC
AU-UA-UA
CG-UA-AU
CG-UA-CG
CG-UA-GC
CG-UA-UA
GC-UA-AU
GC-UA-CG
GC-UA-GC
GC-UA-UA
UA-UA-AU
UA-UA-CG
UA-UA-GC
UA-UA-UA
AU-GC-GU
AU-GC-UG
CG-GC-GU
CG-GC-UG
GC-GC-GU
GC-GC-UG
GU-GC-AU
GU-GC-CG
GU-GC-GC
GU-GC-GU
GU-GC-UA
GU-GC-UG
UA-GC-GU
UA-GC-UG
UG-GC-AU
UG-GC-CG
UG-GC-GC
UG-GC-GU
UG-GC-UA
UG-GC-UG
AU-GU-AU
AU-GU-CG
AU-GU-GC
AU-GU-GU
AU-GU-UA
AU-GU-UG
CG-GU-AU
CG-GU-CG
CG-GU-GC
CG-GU-GU
CG-GU-UA
CG-GU-UG
GC-GU-AU
GC-GU-CG
GC-GU-GC
GC-GU-GU
GC-GU-UA
GC-GU-UG
GU-GU-AU
GU-GU-CG
Dz [Å]
4.5
4.0
3.5
3.0
2.5
2.0
GU-GU-GC
GU-GU-GU
GU-GU-UA
GU-GU-UG
UA-GU-AU
UA-GU-CG
UA-GU-GC
UA-GU-GU
UA-GU-UA
UA-GU-UG
UG-GU-AU
UG-GU-CG
UG-GU-GC
UG-GU-GU
UG-GU-UA
UG-GU-UG
AU-UA-GU
AU-UA-UG
CG-UA-GU
CG-UA-UG
GC-UA-GU
GC-UA-UG
GU-UA-AU
GU-UA-CG
GU-UA-GC
GU-UA-GU
GU-UA-UA
GU-UA-UG
UA-UA-GU
UA-UA-UG
UG-UA-AU
UG-UA-CG
UG-UA-GC
UG-UA-GU
UG-UA-UA
UG-UA-UG
AU-UG-AU
AU-UG-CG
AU-UG-GC
AU-UG-GU
AU-UG-UA
AU-UG-UG
CG-UG-AU
CG-UG-CG
CG-UG-GC
CG-UG-GU
CG-UG-UA
CG-UG-UG
GC-UG-AU
GC-UG-CG
GC-UG-GC
GC-UG-GU
GC-UG-UA
GC-UG-UG
GU-UG-AU
GU-UG-CG
GU-UG-GC
GU-UG-GU
GU-UG-UA
GU-UG-UG
UA-UG-AU
UA-UG-CG
UA-UG-GC
UA-UG-GU
UA-UG-UA
UA-UG-UG
UG-UG-AU
UG-UG-CG
UG-UG-GC
UG-UG-GU
UG-UG-UA
UG-UG-UG

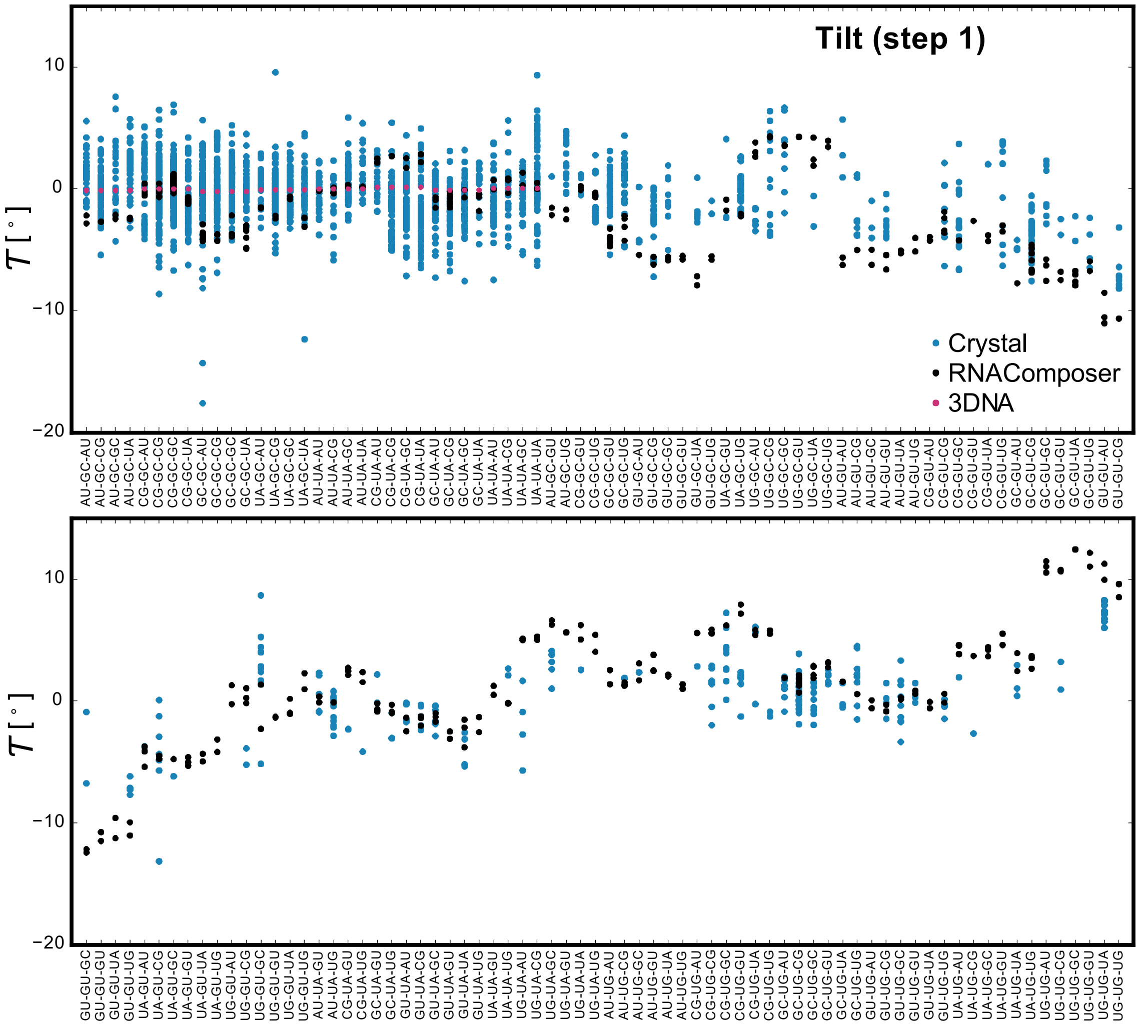
Tilt (step 1)
Crystal
RNAComposer
3DNA
$\mathcal{T}$ [°]
10
0
-10
-20

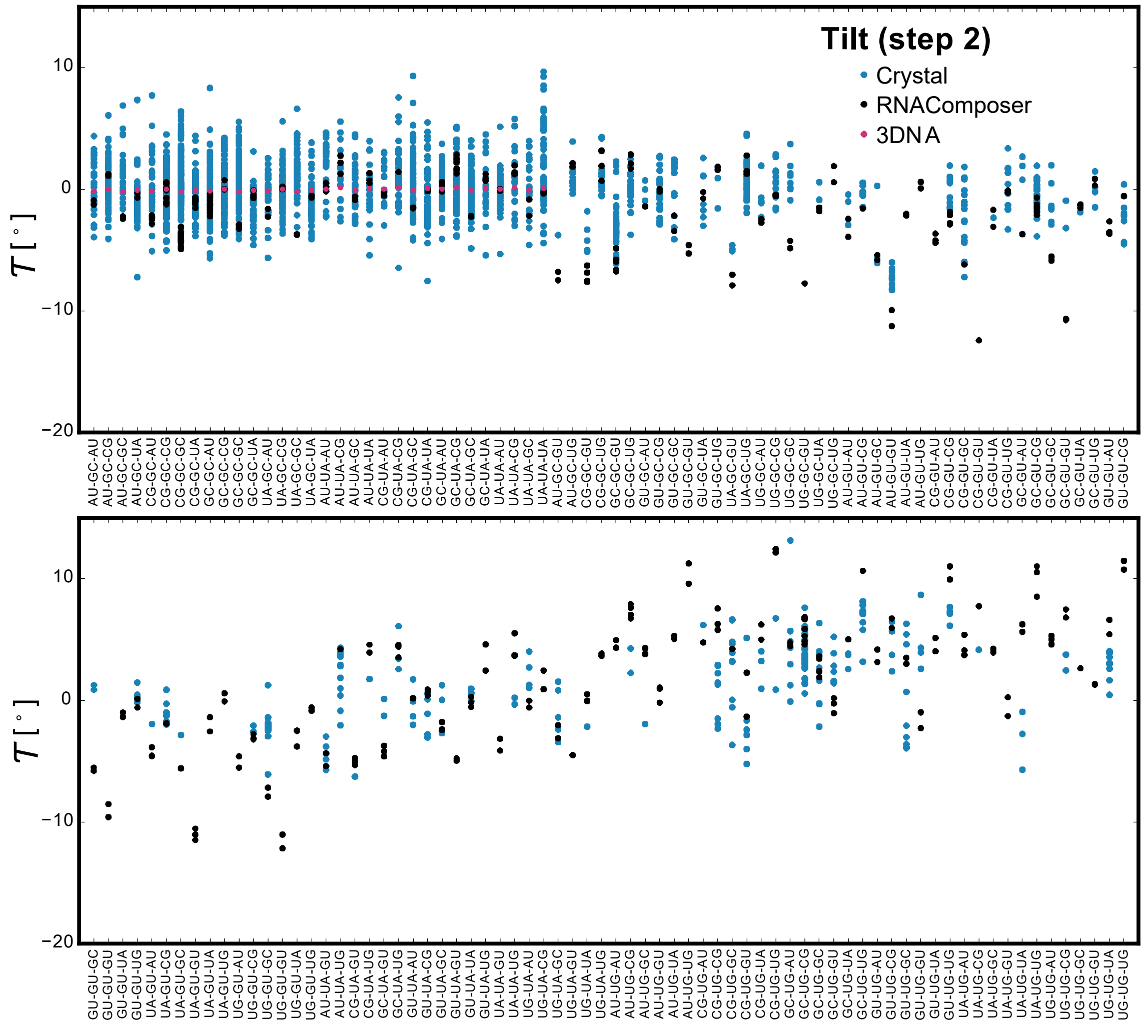
Tilt (step 2)
Crystal
RNAComposer
3DNA

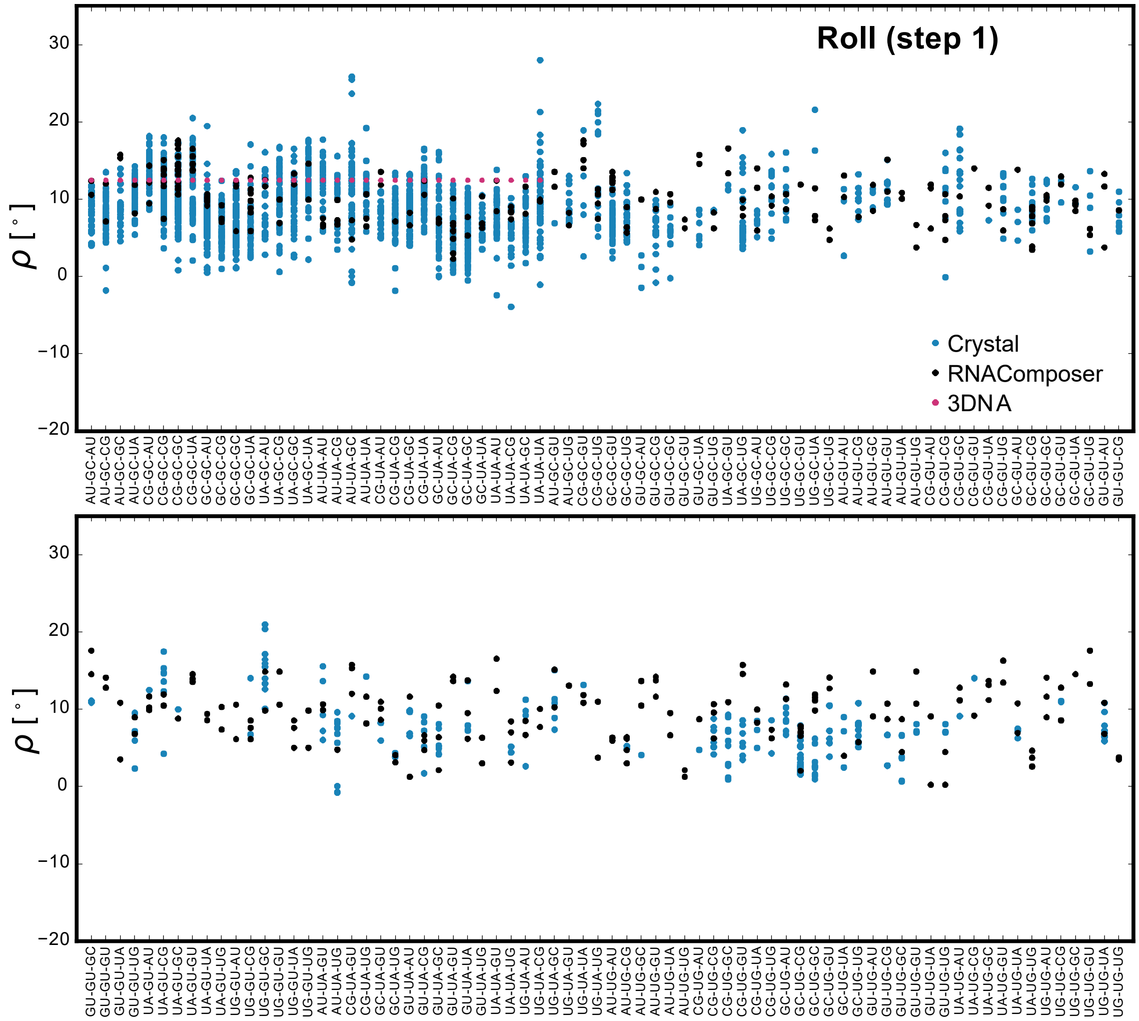
Roll (step 1)
Crystal
RNAComposer
3DNA
ρ [°]
30
20
10
0
−10
−20
AU-GC-AU AU-GC-CG AU-GC-GC AU-GC-UA CG-GC-AU CG-GC-CG CG-GC-GC CG-GC-UA GC-GC-AU GC-GC-CG GC-GC-GC GC-GC-UA UA-GC-AU UA-GC-CG UA-GC-GC UA-GC-UA AU-UA-AU AU-UA-CG AU-UA-GC AU-UA-UA CG-UA-AU CG-UA-CG CG-UA-GC CG-UA-UA GC-UA-AU GC-UA-CG GC-UA-GC GC-UA-UA UA-UA-AU UA-UA-CG UA-UA-GC UA-UA-UA AU-GC-GU AU-GC-UG CG-GC-GU CG-GC-UG GC-GC-GU GC-GC-UG GU-GC-AU GU-GC-CG GU-GC-GC GU-GC-GU GU-GC-UA GU-GC-UG UA-GC-GU UA-GC-UG UG-GC-AU UG-GC-CG UG-GC-GC UG-GC-GU UG-GC-UA UG-GC-UG AU-GU-AU AU-GU-CG AU-GU-GC AU-GU-GU AU-GU-UA AU-GU-UG CG-GU-AU CG-GU-CG CG-GU-GC CG-GU-GU CG-GU-UA CG-GU-UG GC-GU-AU GC-GU-CG GC-GU-GC GC-GU-GU GC-GU-UA GC-GU-UG GU-GU-AU GU-GU-CG
ρ [°]
30
20
10
0
−10
−20
GU-GU-GC GU-GU-GU GU-GU-UA GU-GU-UG UA-GU-AU UA-GU-CG UA-GU-GC UA-GU-GU UA-GU-UA UA-GU-UG UG-GU-AU UG-GU-CG UG-GU-GC UG-GU-GU UG-GU-UA UG-GU-UG AU-UA-GU AU-UA-UG CG-UA-GU CG-UA-UG GC-UA-GU GC-UA-UG GU-UA-AU GU-UA-CG GU-UA-GC GU-UA-GU GU-UA-UA GU-UA-UG UA-UA-GU UA-UA-UG UG-UA-AU UG-UA-CG UG-UA-GC UG-UA-GU UG-UA-UA UG-UA-UG AU-UG-AU AU-UG-CG AU-UG-GC AU-UG-GU AU-UG-UA AU-UG-UG CG-UG-AU CG-UG-CG CG-UG-GC CG-UG-GU CG-UG-UA CG-UG-UG GC-UG-AU GC-UG-CG GC-UG-GC GC-UG-GU GC-UG-UA GC-UG-UG GU-UG-AU GU-UG-CG GU-UG-GC GU-UG-GU GU-UG-UA GU-UG-UG UA-UG-AU UA-UG-CG UA-UG-GC UA-UG-GU UA-UG-UA UA-UG-UG UG-UG-AU UG-UG-CG UG-UG-GC UG-UG-GU UG-UG-UA UG-UG-UG

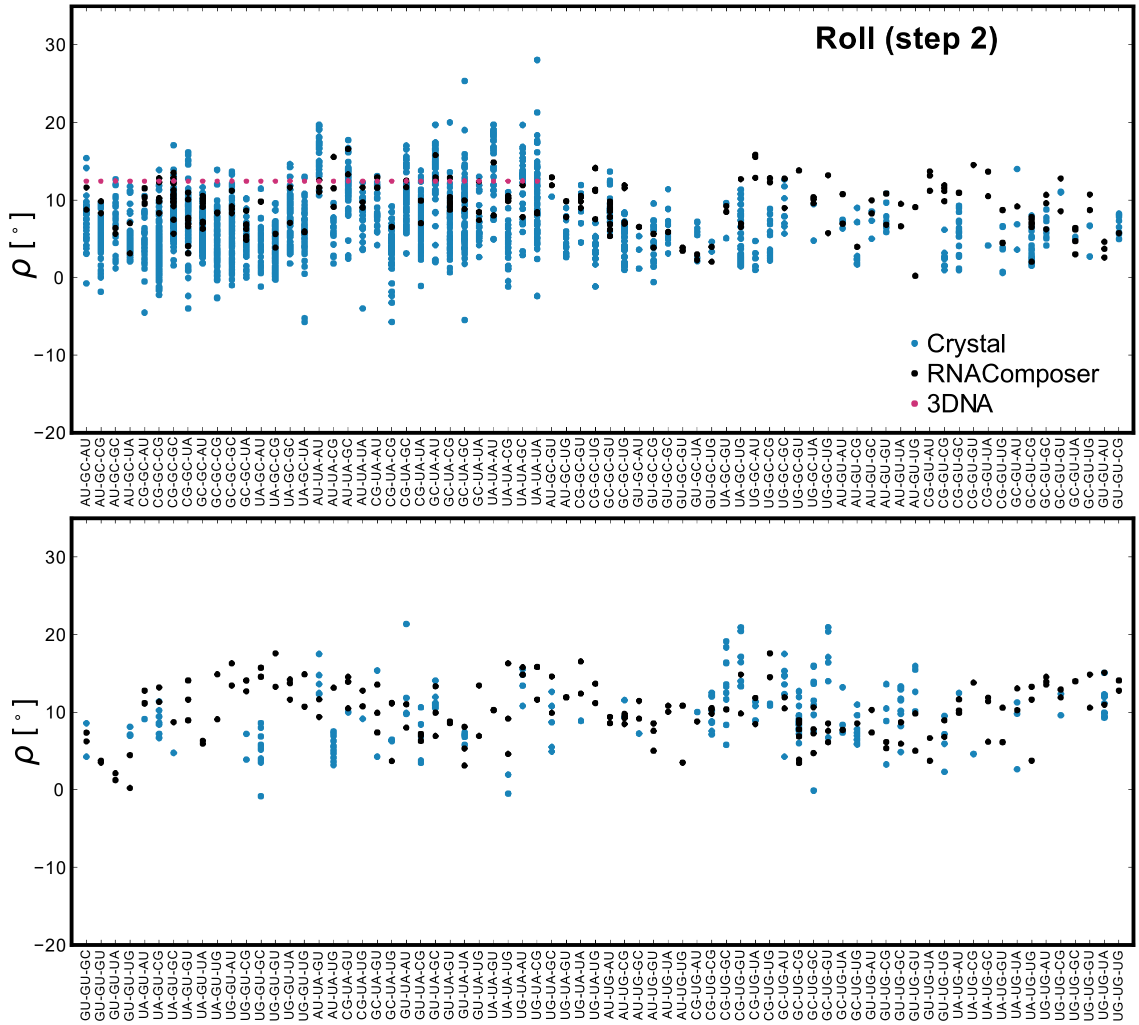

Roll (step 2)
Crystal
RNAComposer
3DNA
ρ [°]
30
20
10
0
−10
−20

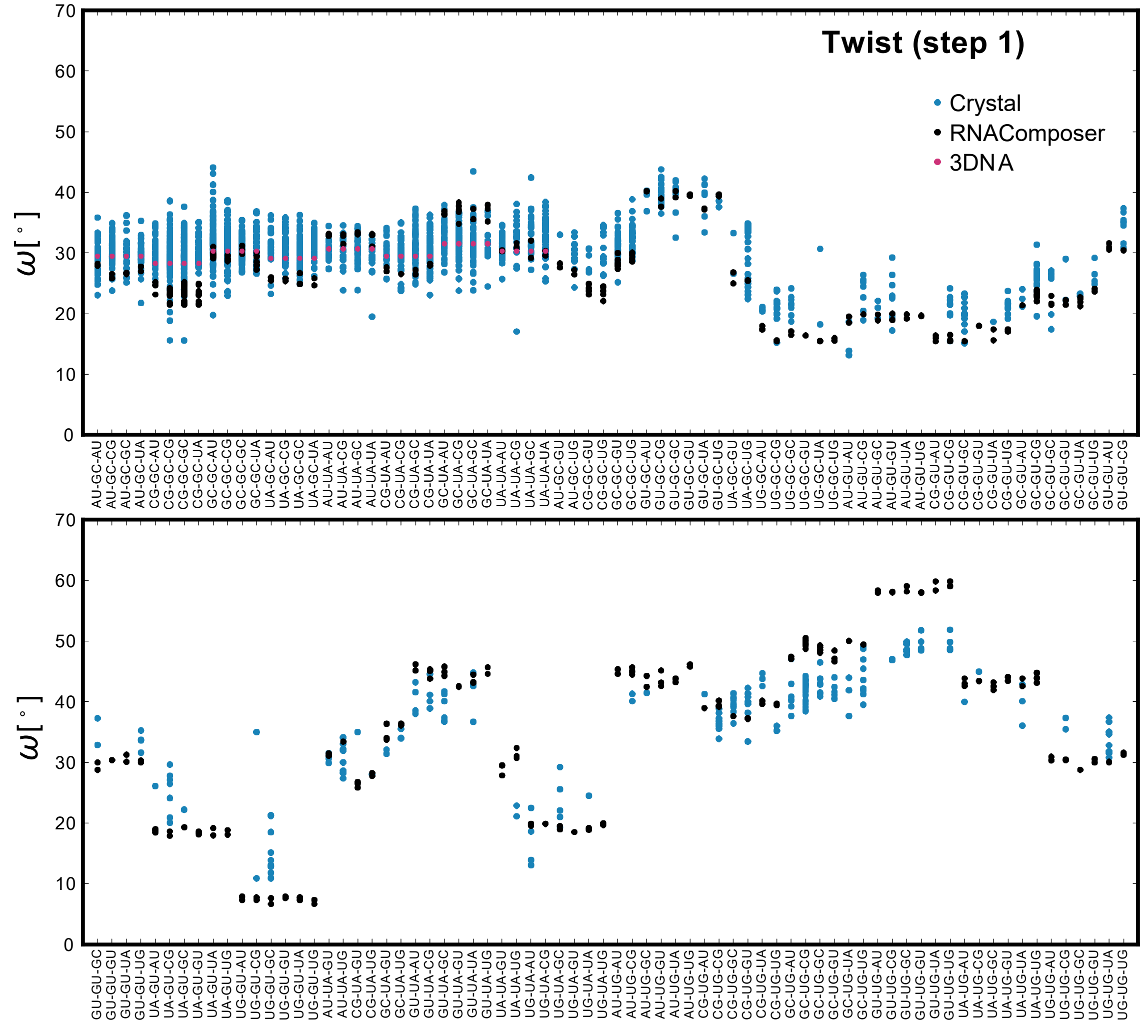
Twist (step 1)
Crystal
RNAComposer
3DNA
ω[°]
0
10
20
30
40
50
60
70
AU-GC-AU
AU-GC-CG
AU-GC-GC
AU-GC-UA
CG-GC-AU
CG-GC-CG
CG-GC-GC
CG-GC-UA
GC-GC-AU
GC-GC-CG
GC-GC-GC
GC-GC-UA
UA-GC-AU
UA-GC-CG
UA-GC-GC
UA-GC-UA
AU-UA-AU
AU-UA-CG
AU-UA-GC
AU-UA-UA
CG-UA-AU
CG-UA-CG
CG-UA-GC
CG-UA-UA
GC-UA-AU
GC-UA-CG
GC-UA-GC
GC-UA-UA
UA-UA-AU
UA-UA-CG
UA-UA-GC
UA-UA-UA
AU-GC-GU
AU-GC-UG
CG-GC-GU
CG-GC-UG
GC-GC-GU
GC-GC-UG
GU-GC-AU
GU-GC-CG
GU-GC-GC
GU-GC-GU
GU-GC-UA
GU-GC-UG
UA-GC-GU
UA-GC-UG
UG-GC-AU
UG-GC-CG
UG-GC-GC
UG-GC-GU
UG-GC-UA
UG-GC-UG
AU-GU-AU
AU-GU-CG
AU-GU-GC
AU-GU-GU
AU-GU-UA
AU-GU-UG
CG-GU-AU
CG-GU-CG
CG-GU-GC
CG-GU-GU
CG-GU-UA
CG-GU-UG
GC-GU-AU
GC-GU-CG
GC-GU-GC
GC-GU-GU
GC-GU-UA
GC-GU-UG
GU-GU-AU
GU-GU-CG
ω[°]
0
10
20
30
40
50
60
70
GU-GU-GC
GU-GU-GU
GU-GU-UA
GU-GU-UG
UA-GU-AU
UA-GU-CG
UA-GU-GC
UA-GU-GU
UA-GU-UA
UA-GU-UG
UG-GU-AU
UG-GU-CG
UG-GU-GC
UG-GU-GU
UG-GU-UA
UG-GU-UG
AU-UA-GU
AU-UA-UG
CG-UA-GU
CG-UA-UG
GC-UA-GU
GC-UA-UG
GU-UA-AU
GU-UA-CG
GU-UA-GC
GU-UA-GU
GU-UA-UA
GU-UA-UG
UA-UA-GU
UA-UA-UG
UG-UA-AU
UG-UA-CG
UG-UA-GC
UG-UA-GU
UG-UA-UA
UG-UA-UG
AU-UG-AU
AU-UG-CG
AU-UG-GC
AU-UG-GU
AU-UG-UA
AU-UG-UG
CG-UG-AU
CG-UG-CG
CG-UG-GC
CG-UG-GU
CG-UG-UA
CG-UG-UG
GC-UG-AU
GC-UG-CG
GC-UG-GC
GC-UG-GU
GC-UG-UA
GC-UG-UG
GU-UG-AU
GU-UG-CG
GU-UG-GC
GU-UG-GU
GU-UG-UA
GU-UG-UG
UA-UG-AU
UA-UG-CG
UA-UG-GC
UA-UG-GU
UA-UG-UA
UA-UG-UG
UG-UG-AU
UG-UG-CG
UG-UG-GC
UG-UG-GU
UG-UG-UA
UG-UG-UG

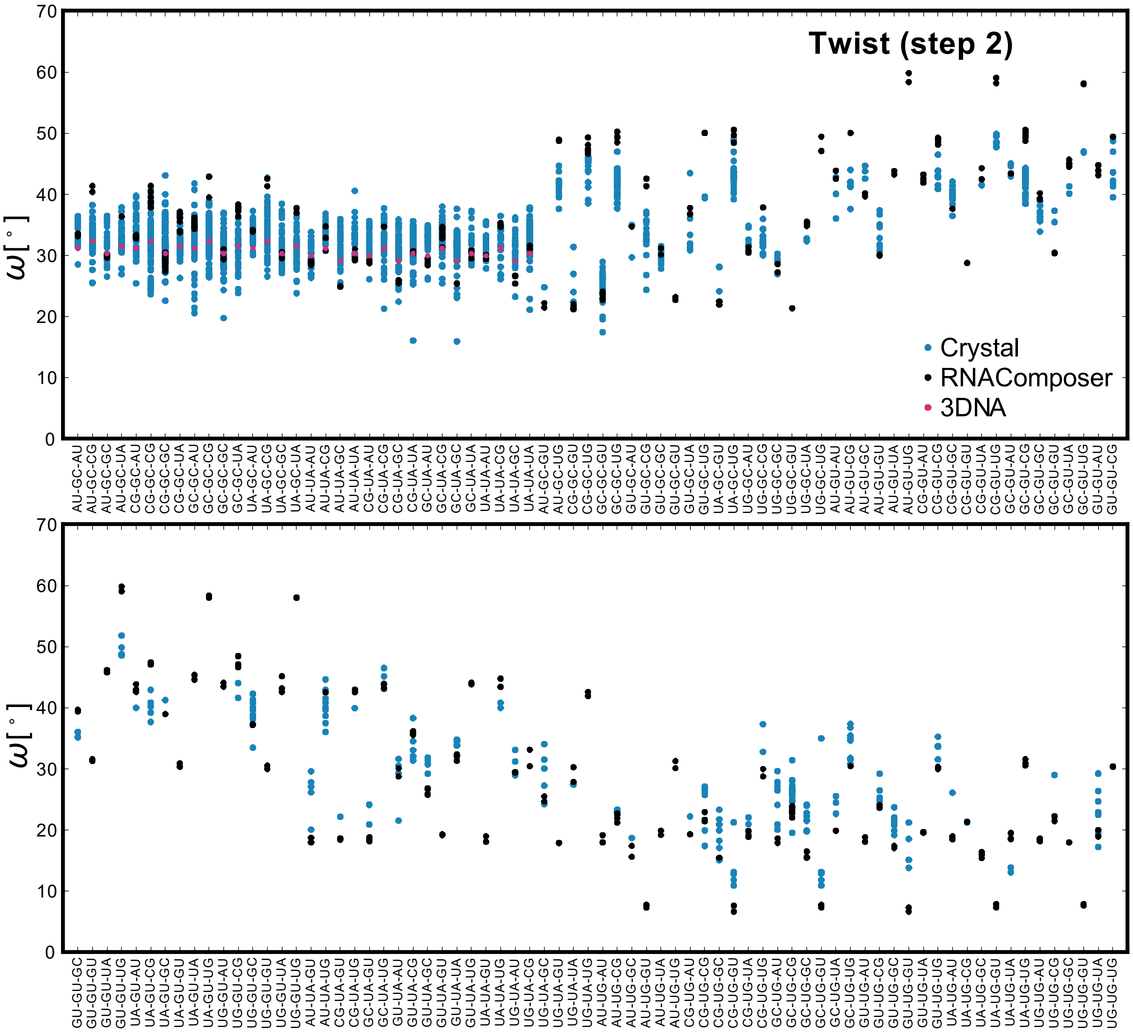
Twist (step 2)
Crystal
RNAComposer
3DNA
ω[°]

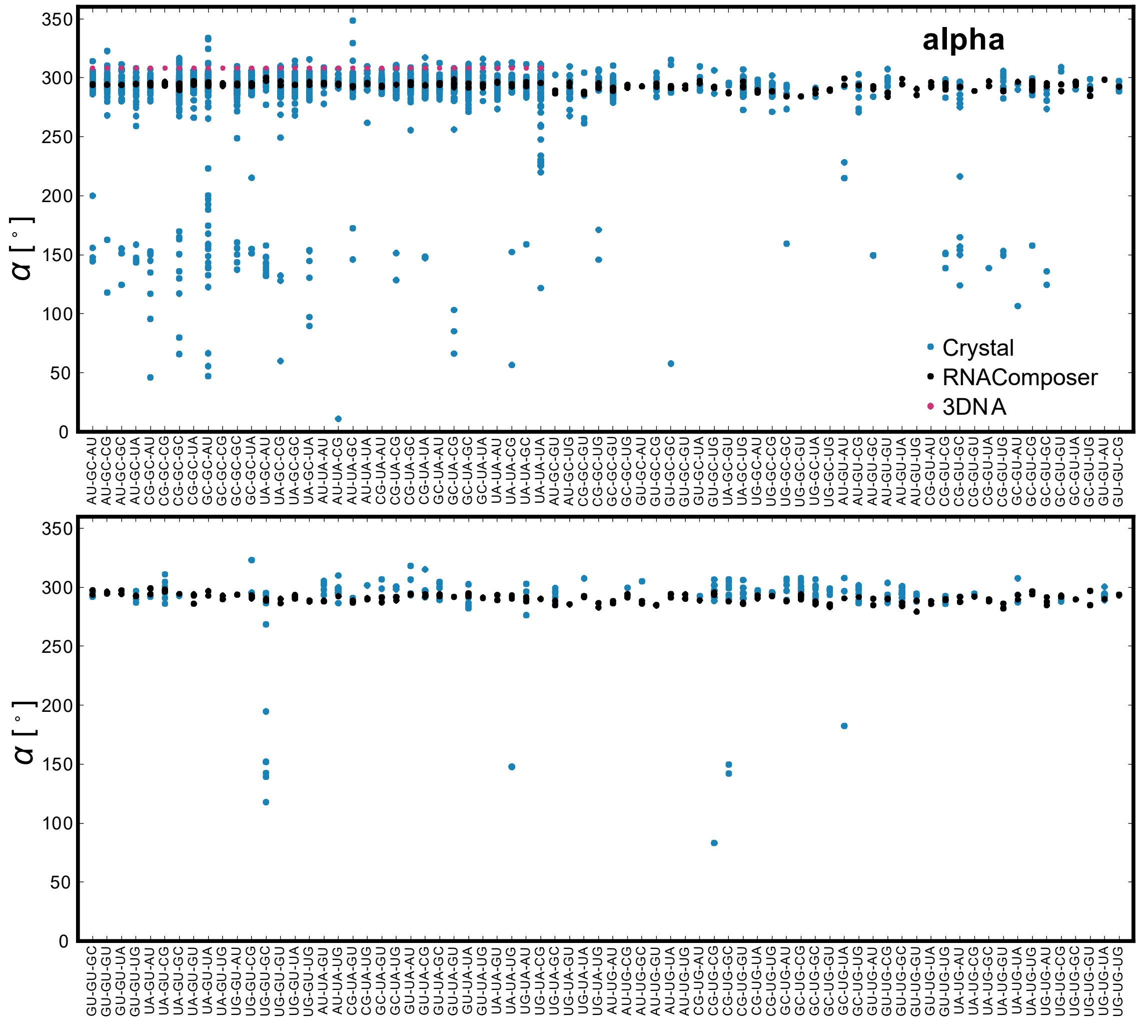

alpha
Crystal
RNAComposer
3DNA
α [°]
α [°]

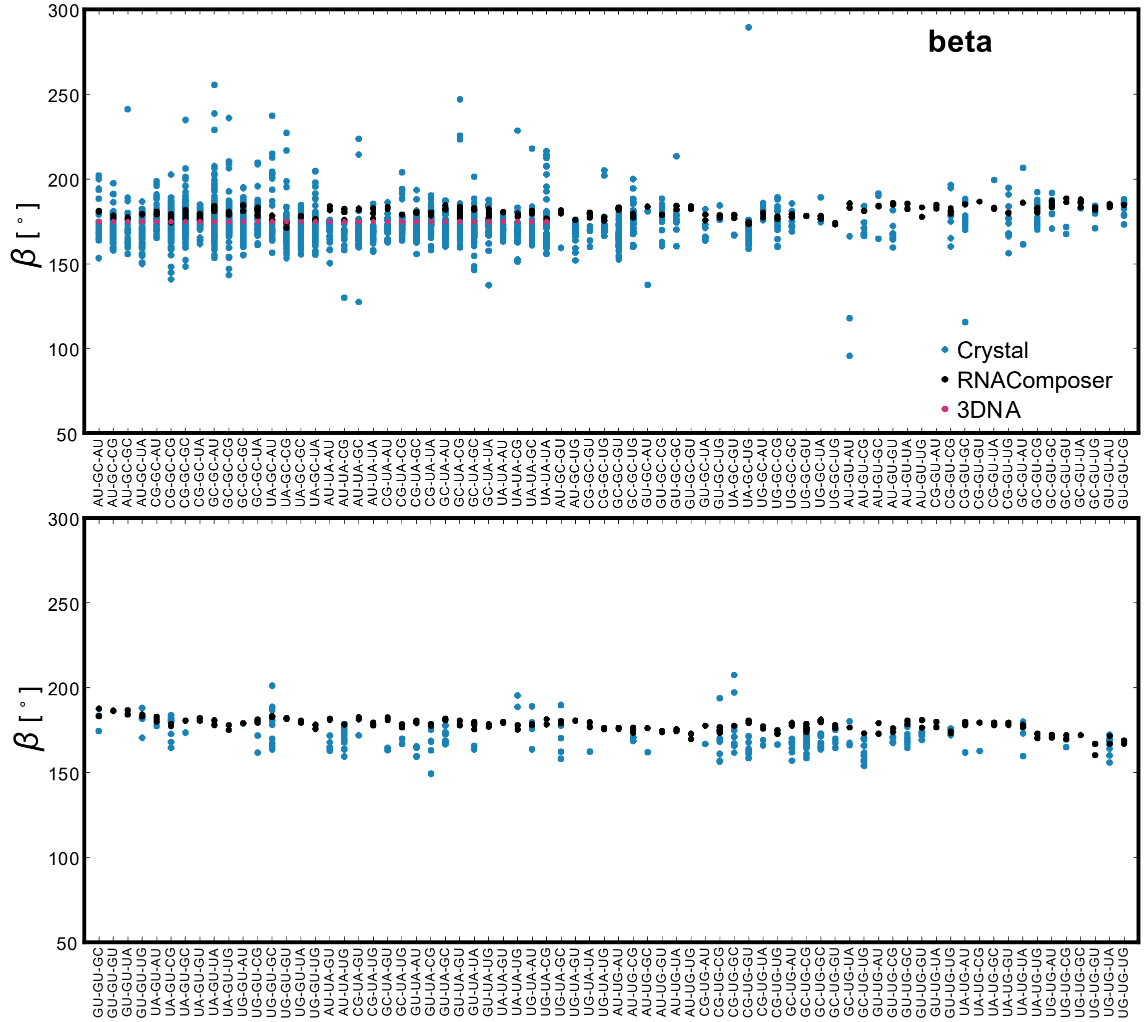
beta
Crystal
RNAComposer
3DNA
β [°]
300
250
200
150
100
50

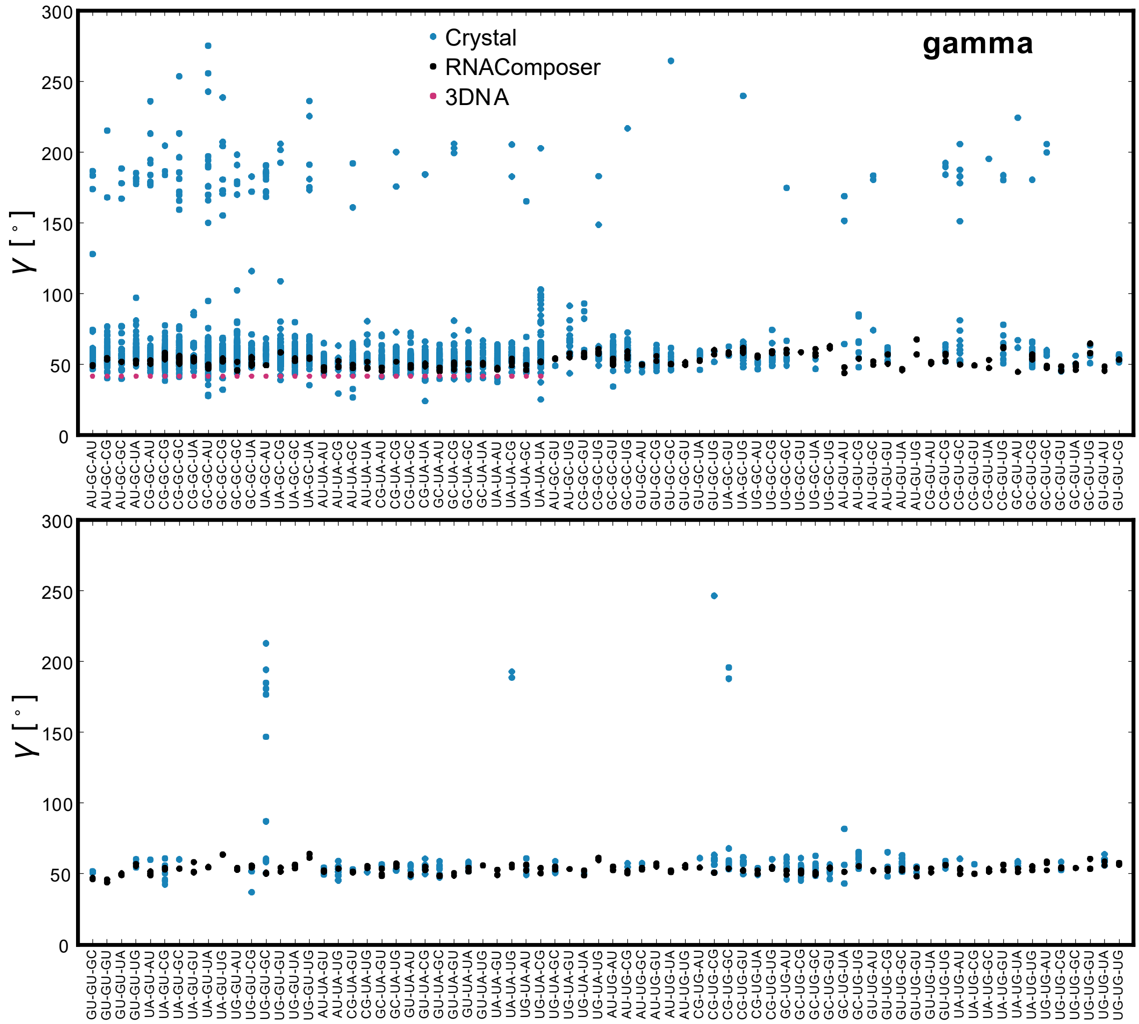

gamma
Crystal
RNAComposer
3DNA
γ [°]
0
50
100
150
200
250
300
AU-GC-AU
AU-GC-CG
AU-GC-GC
AU-GC-UA
CG-GC-AU
CG-GC-CG
CG-GC-GC
CG-GC-UA
GC-GC-AU
GC-GC-CG
GC-GC-GC
GC-GC-UA
UA-GC-AU
UA-GC-CG
UA-GC-GC
UA-GC-UA
AU-UA-AU
AU-UA-CG
AU-UA-GC
AU-UA-UA
CG-UA-AU
CG-UA-CG
CG-UA-GC
CG-UA-UA
GC-UA-AU
GC-UA-CG
GC-UA-GC
GC-UA-UA
UA-UA-AU
UA-UA-CG
UA-UA-GC
UA-UA-UA
AU-GC-GU
AU-GC-UG
CG-GC-GU
CG-GC-UG
GC-GC-GU
GC-GC-UG
GU-GC-AU
GU-GC-CG
GU-GC-GC
GU-GC-GU
GU-GC-UA
GU-GC-UG
UA-GC-GU
UA-GC-UG
UG-GC-AU
UG-GC-CG
UG-GC-GC
UG-GC-GU
UG-GC-UA
UG-GC-UG
AU-GU-AU
AU-GU-CG
AU-GU-GC
AU-GU-GU
AU-GU-UA
AU-GU-UG
CG-GU-AU
CG-GU-CG
CG-GU-GC
CG-GU-GU
CG-GU-UA
CG-GU-UG
GC-GU-AU
GC-GU-CG
GC-GU-GC
GC-GU-GU
GC-GU-UA
GC-GU-UG
GU-GU-AU
GU-GU-CG
γ [°]
0
50
100
150
200
250
300
GU-GU-GC
GU-GU-GU
GU-GU-UA
GU-GU-UG
UA-GU-AU
UA-GU-CG
UA-GU-GC
UA-GU-GU
UA-GU-UA
UA-GU-UG
UG-GU-AU
UG-GU-CG
UG-GU-GC
UG-GU-GU
UG-GU-UA
UG-GU-UG
AU-UA-GU
AU-UA-UG
CG-UA-GU
CG-UA-UG
GC-UA-GU
GC-UA-UG
GU-UA-AU
GU-UA-CG
GU-UA-GC
GU-UA-GU
GU-UA-UA
GU-UA-UG
UA-UA-GU
UA-UA-UG
UG-UA-AU
UG-UA-CG
UG-UA-GC
UG-UA-GU
UG-UA-UA
UG-UA-UG
AU-UG-AU
AU-UG-CG
AU-UG-GC
AU-UG-GU
AU-UG-UA
AU-UG-UG
CG-UG-AU
CG-UG-CG
CG-UG-GC
CG-UG-GU
CG-UG-UA
CG-UG-UG
GC-UG-AU
GC-UG-CG
GC-UG-GC
GC-UG-GU
GC-UG-UA
GC-UG-UG
GU-UG-AU
GU-UG-CG
GU-UG-GC
GU-UG-GU
GU-UG-UA
GU-UG-UG
UA-UG-AU
UA-UG-CG
UA-UG-GC
UA-UG-GU
UA-UG-UA
UA-UG-UG
UG-UG-AU
UG-UG-CG
UG-UG-GC
UG-UG-GU
UG-UG-UA
UG-UG-UG

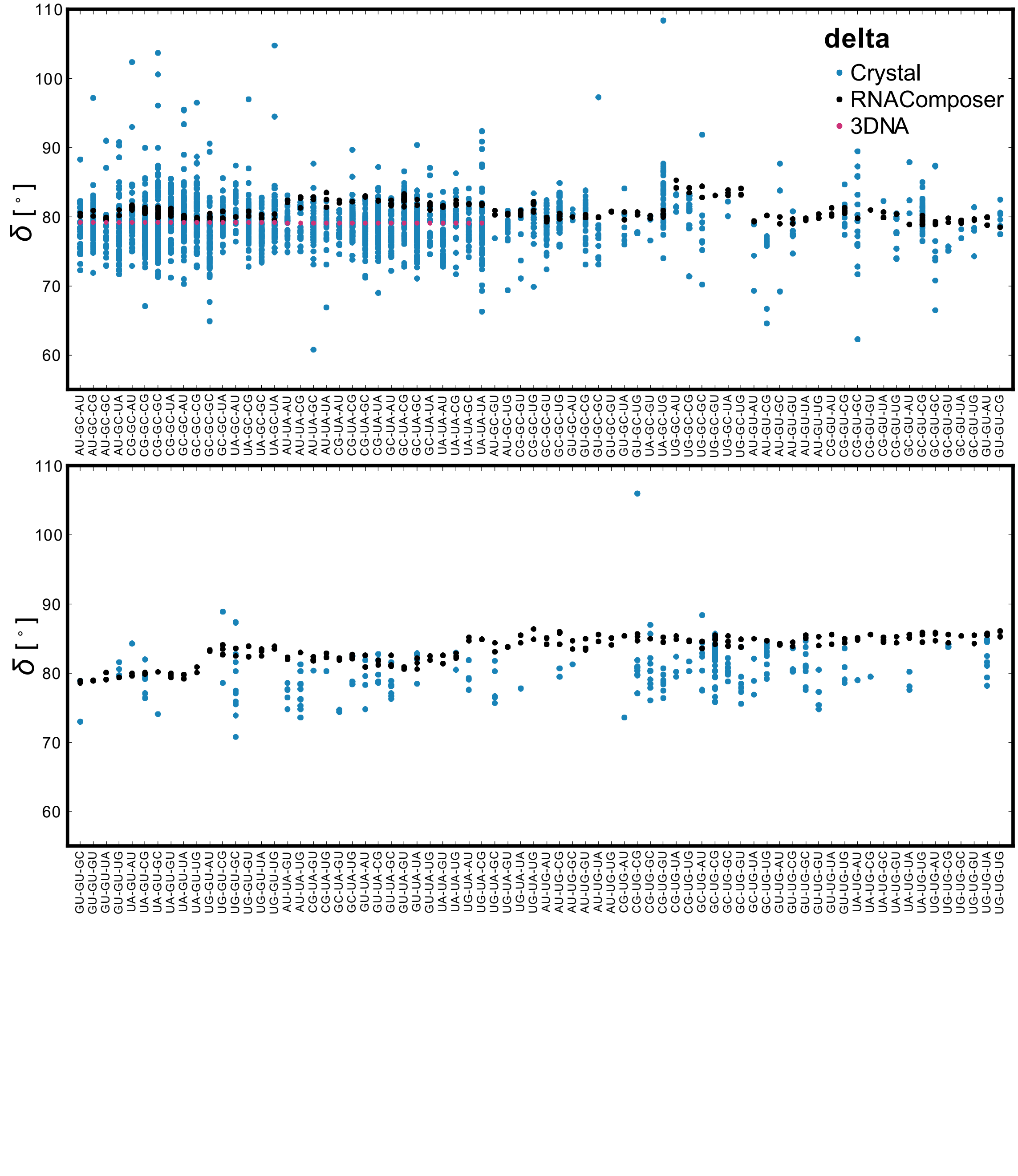
delta
Crystal
RNAComposer
3DNA
δ [°]

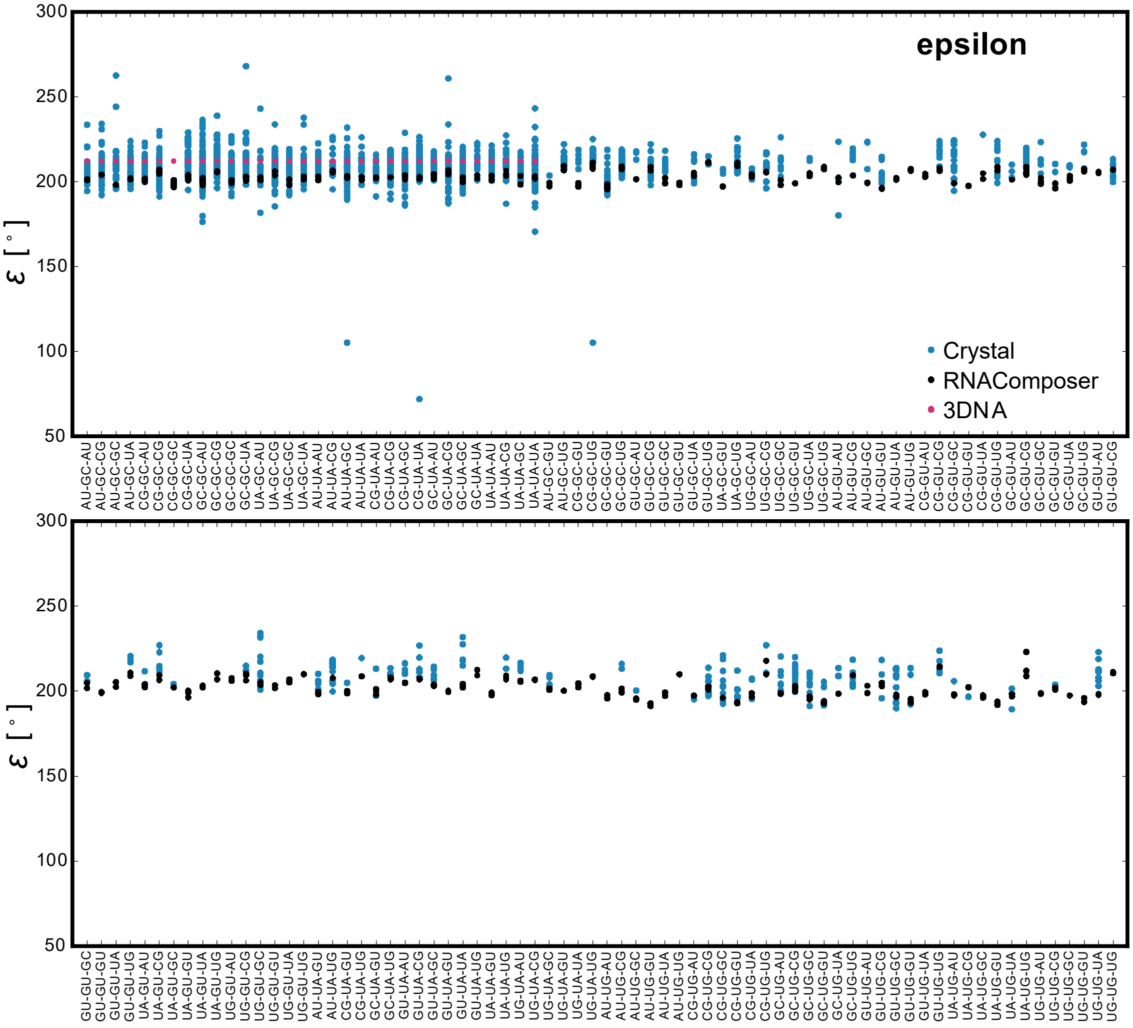
epsilon
ε [°]
300
250
200
150
100
50
Crystal
RNAComposer
3DNA
ε [°]

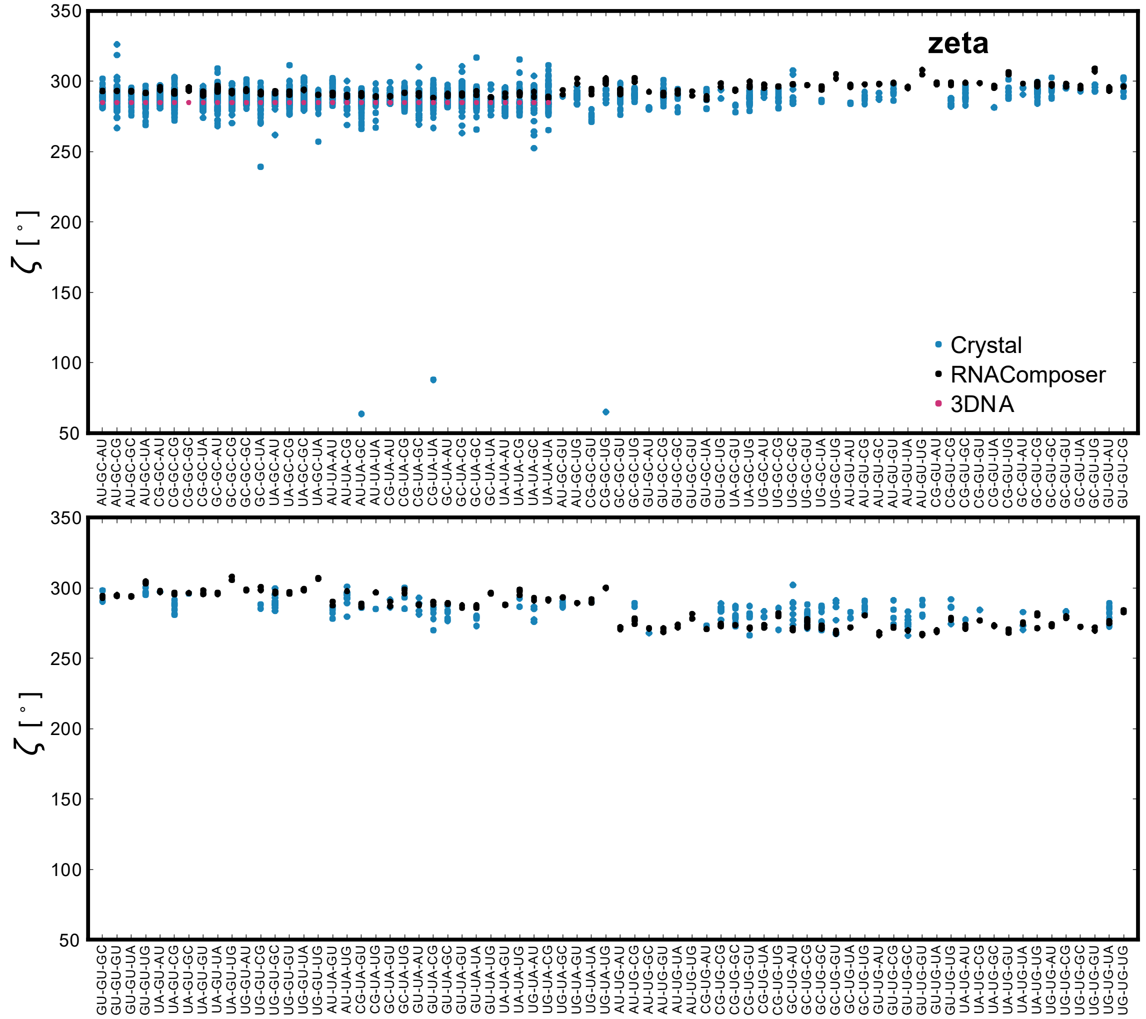
zeta
Crystal
RNAComposer
3DNA
ζ [°]
350
300
250
200
150
100
50

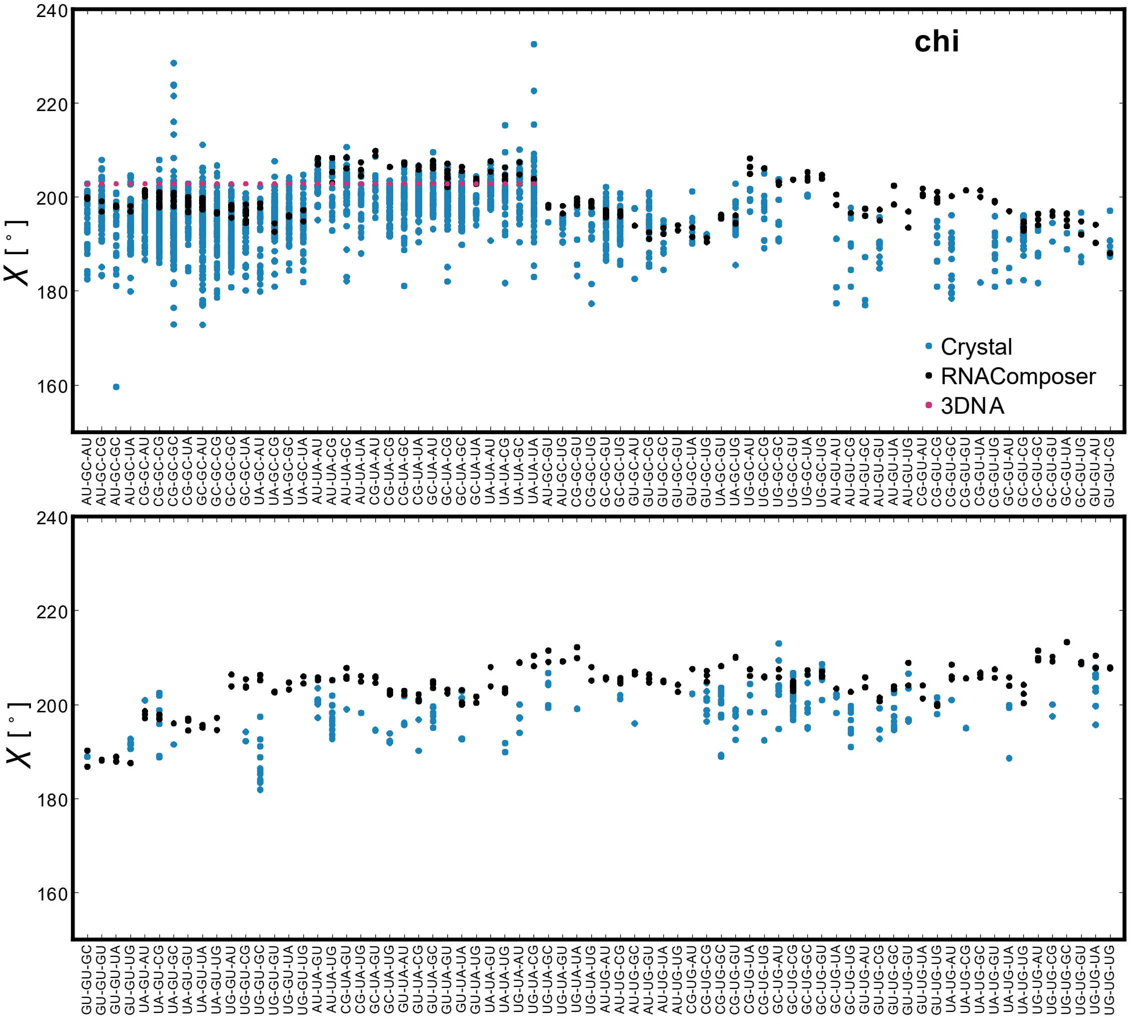
chi
Crystal
RNAComposer
3DNA
χ [°]
240
220
200
180
160

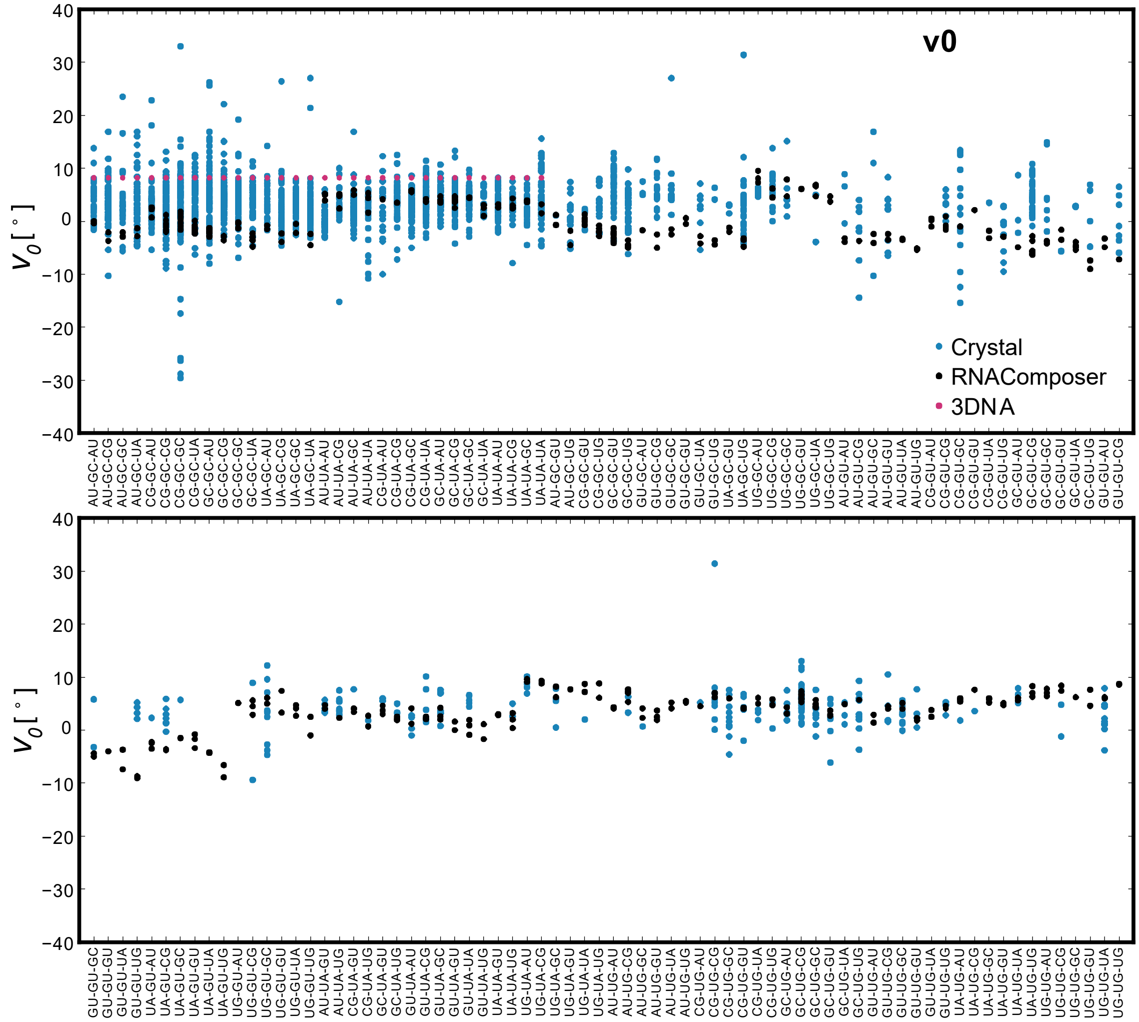
v0
Crystal
RNAComposer
3DNA
V_0 [°]

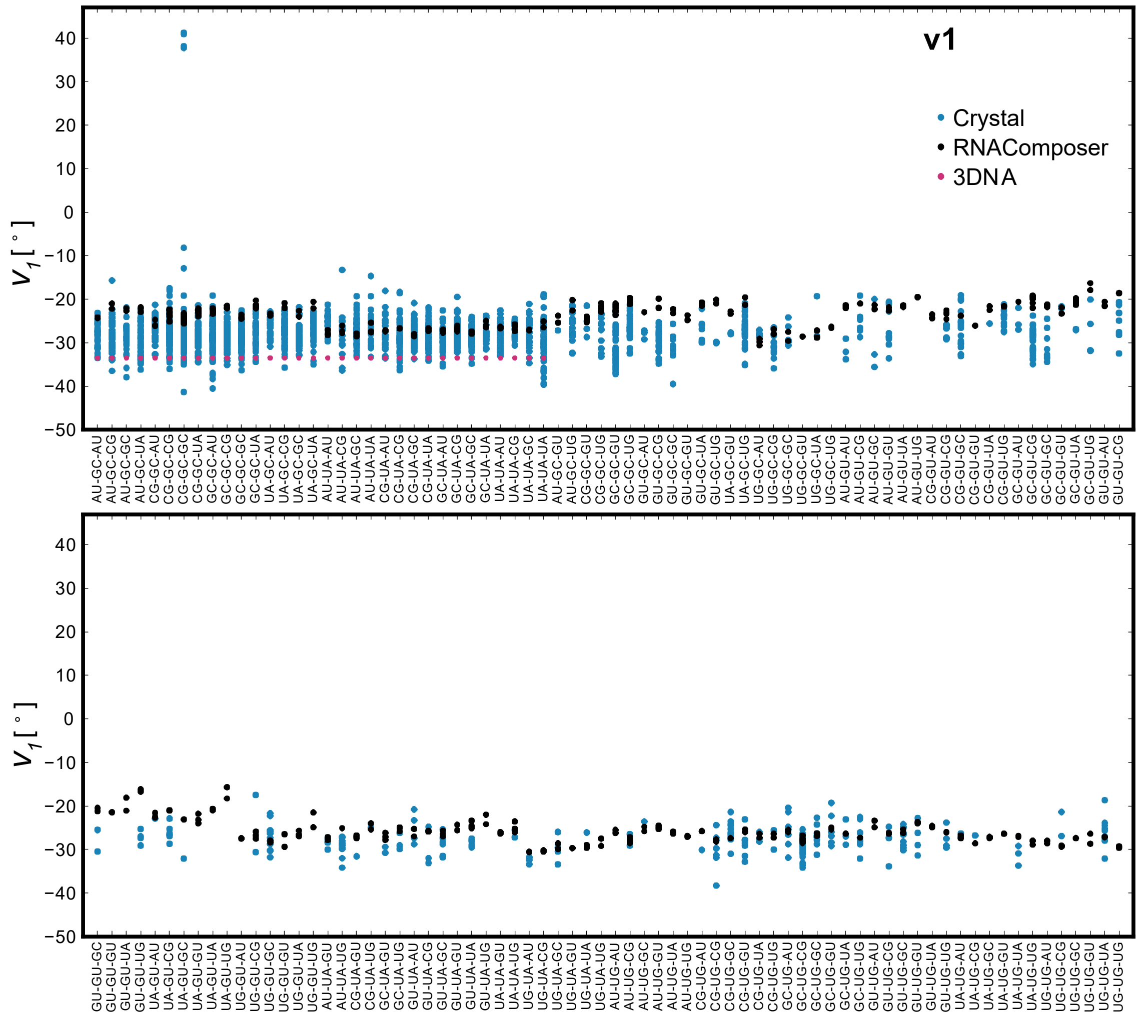
v1
Crystal
RNAComposer
3DNA
V_1 [°]
40
30
20
10
0
-10
-20
-30
-40
-50
V_1 [°]
40
30
20
10
0
-10
-20
-30
-40
-50

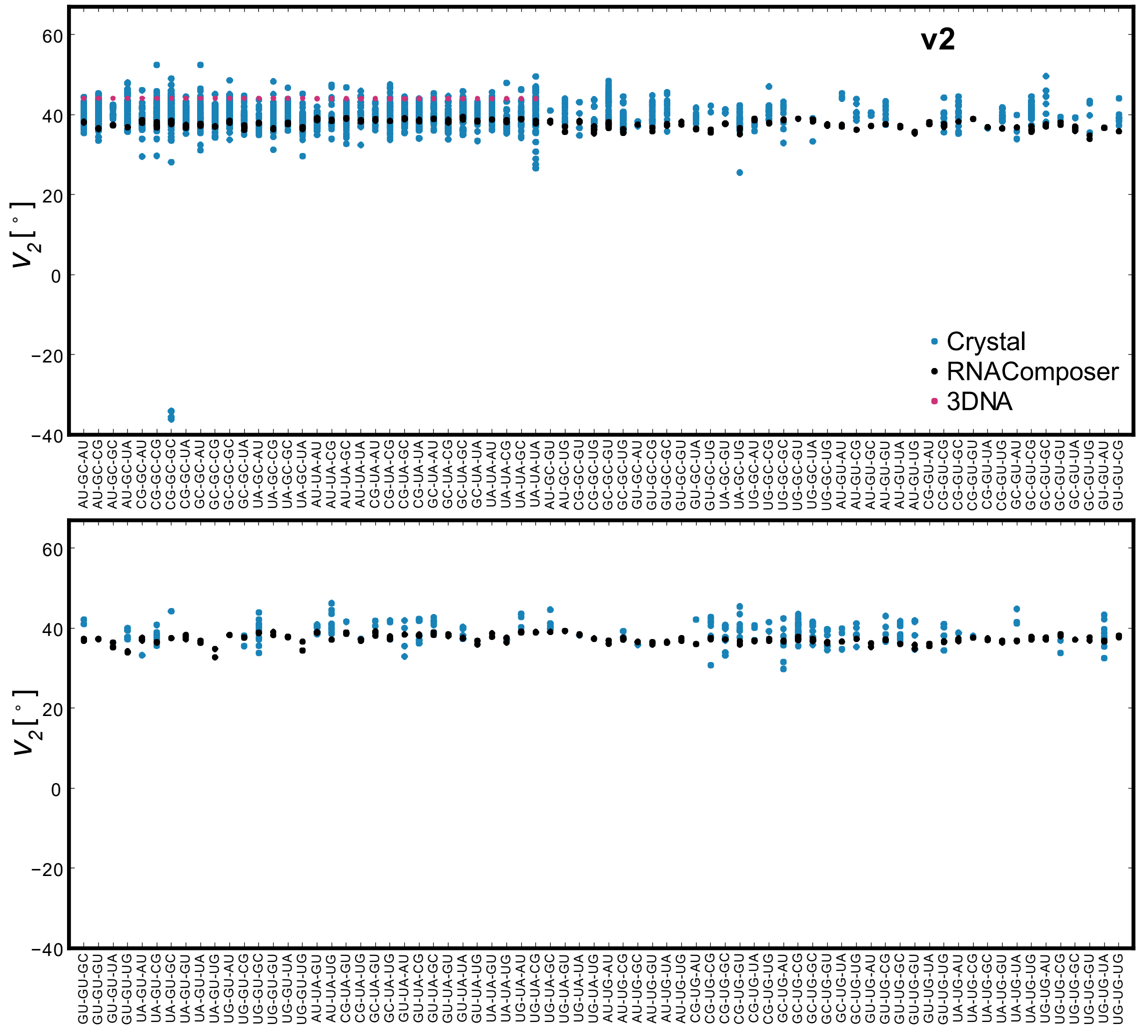
v2
Crystal
RNAComposer
3DNA
v_2 [°]
60
40
20
0
−20
−40
AU-GC-AU AU-GC-CG AU-GC-GC AU-GC-UA CG-GC-AU CG-GC-CG CG-GC-GC CG-GC-UA GC-GC-AU GC-GC-CG GC-GC-GC GC-GC-UA UA-GC-AU UA-GC-CG UA-GC-GC UA-GC-UA AU-UA-AU AU-UA-CG AU-UA-GC AU-UA-UA CG-UA-AU CG-UA-CG CG-UA-GC CG-UA-UA GC-UA-AU GC-UA-CG GC-UA-GC GC-UA-UA UA-UA-AU UA-UA-CG UA-UA-GC UA-UA-UA AU-GC-GU AU-GC-UG CG-GC-GU CG-GC-UG GC-GC-GU GC-GC-UG GU-GC-AU GU-GC-CG GU-GC-GC GU-GC-GU GU-GC-UA GU-GC-UG UA-GC-GU UA-GC-UG UG-GC-AU UG-GC-CG UG-GC-GC UG-GC-GU UG-GC-UA UG-GC-UG AU-GU-AU AU-GU-CG AU-GU-GC AU-GU-GU AU-GU-UA AU-GU-UG CG-GU-AU CG-GU-CG CG-GU-GC CG-GU-GU CG-GU-UA CG-GU-UG GC-GU-AU GC-GU-CG GC-GU-GC GC-GU-GU GC-GU-UA GC-GU-UG GU-GU-AU GU-GU-CG
v_2 [°]
60
40
20
0
−20
−40
GU-GU-GC GU-GU-GU GU-GU-UA GU-GU-UG UA-GU-AU UA-GU-CG UA-GU-GC UA-GU-GU UA-GU-UA UA-GU-UG UG-GU-AU UG-GU-CG UG-GU-GC UG-GU-GU UG-GU-UA UG-GU-UG AU-UA-GU AU-UA-UG CG-UA-GU CG-UA-UG GC-UA-GU GC-UA-UG GU-UA-AU GU-UA-CG GU-UA-GC GU-UA-GU GU-UA-UA GU-UA-UG UA-UA-GU UA-UA-UG UG-UA-AU UG-UA-CG UG-UA-GC UG-UA-GU UG-UA-UA UG-UA-UG AU-UG-AU AU-UG-CG AU-UG-GC AU-UG-GU AU-UG-UA AU-UG-UG CG-UG-AU CG-UG-CG CG-UG-GC CG-UG-GU CG-UG-UA CG-UG-UG GC-UG-AU GC-UG-CG GC-UG-GC GC-UG-GU GC-UG-UA GC-UG-UG GU-UG-AU GU-UG-CG GU-UG-GC GU-UG-GU GU-UG-UA GU-UG-UG UA-UG-AU UA-UG-CG UA-UG-GC UA-UG-GU UA-UG-UA UA-UG-UG UG-UG-AU UG-UG-CG UG-UG-GC UG-UG-GU UG-UG-UA UG-UG-UG

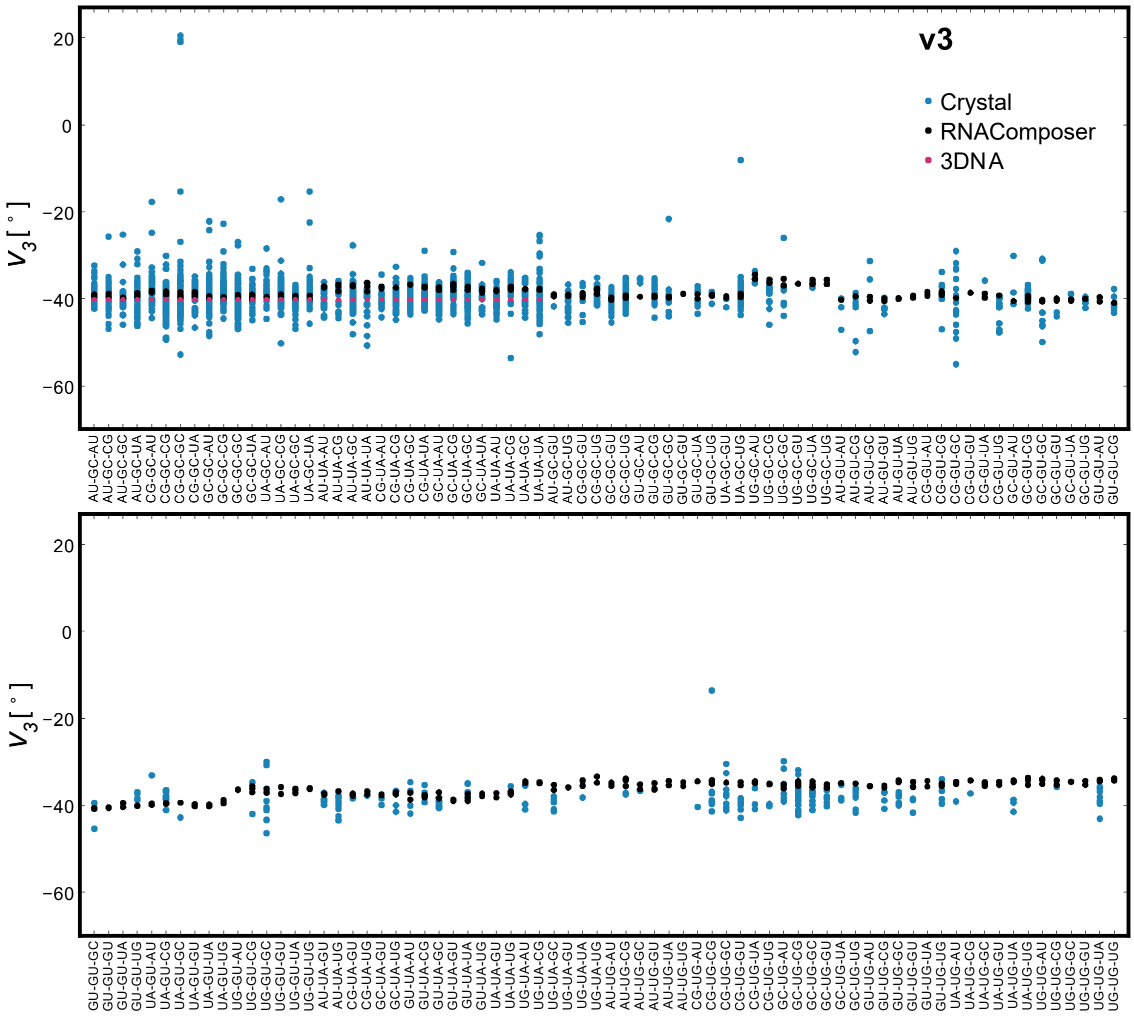
v3
Crystal
RNAComposer
3DNA
V_3[°]
20
0
-20
-40
-60

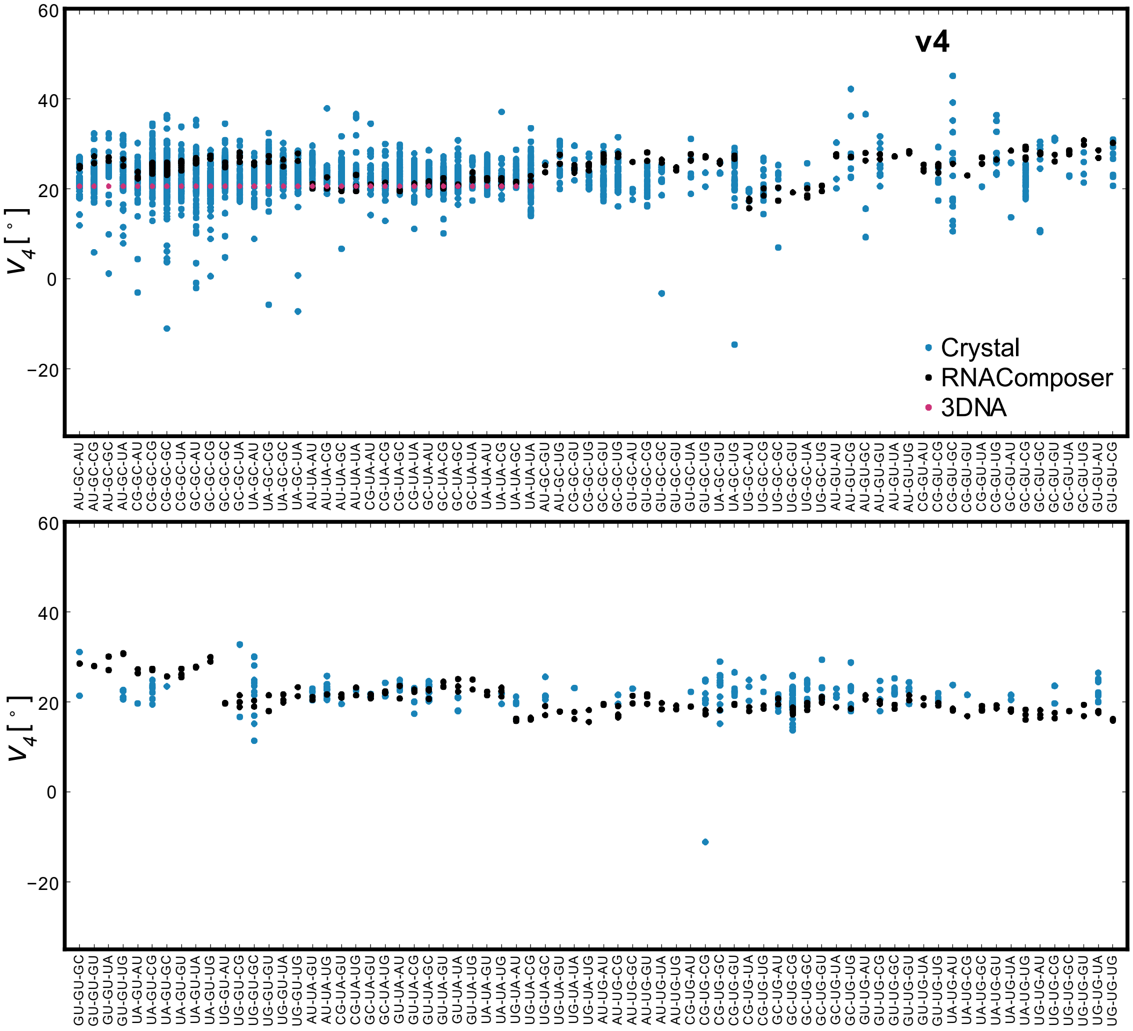
v4
Crystal
RNAComposer
3DNA
60
40
20
0
−20

Figure S9. RC shifts calculated using 3DNA structures (magenta), RNAComposer structures (black) and crystal structures (dark blue). For each BP-triplet, the ^{15}N and $^{1}H^{N}$ chemical shifts are shown in the upper and the lower panels, respectively.

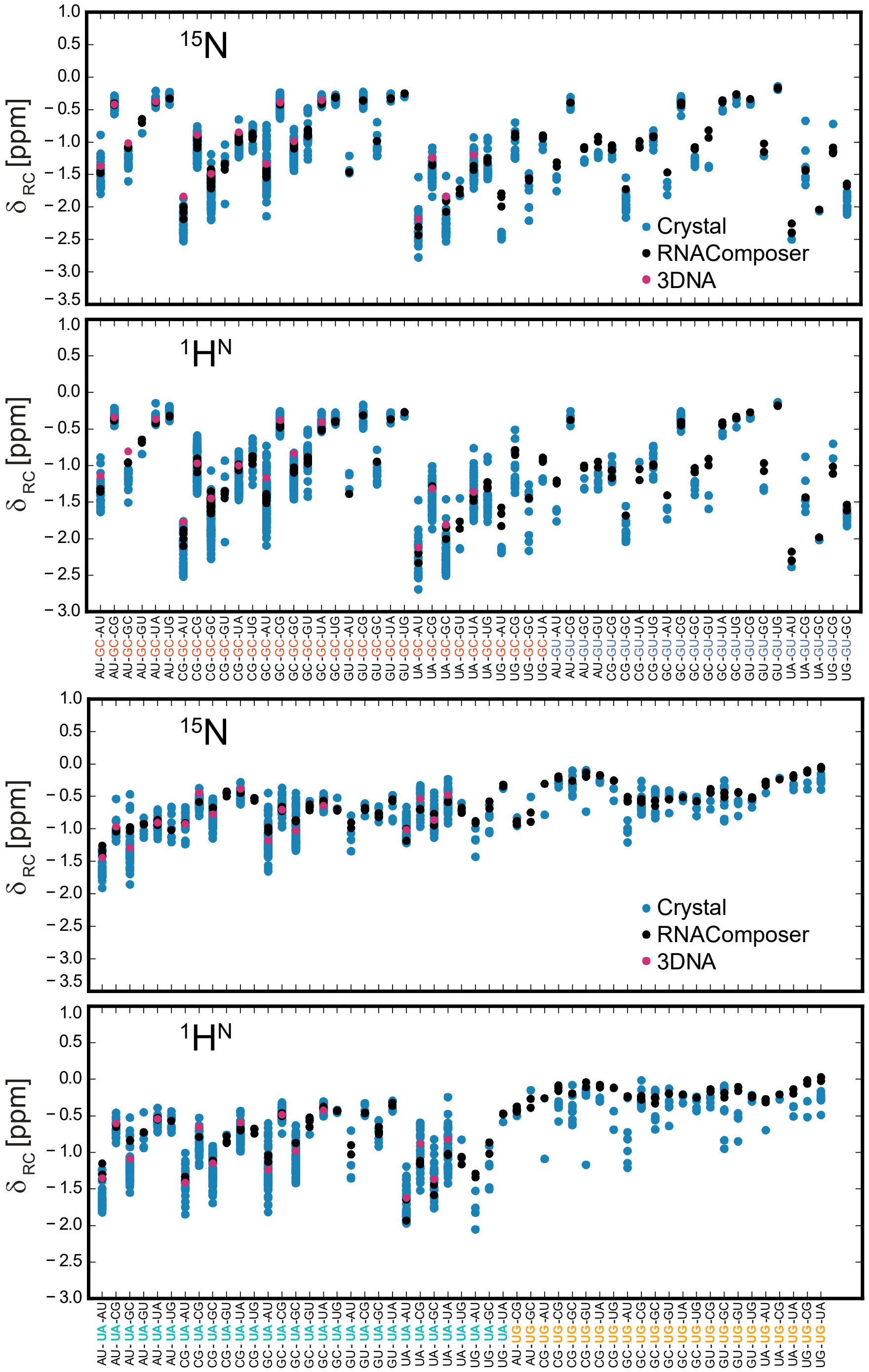

Figure S10. The imino chemical shift map produced by the semi-empirical method. (a) Correlation between chemical shifts calculated by RC (calibrated ^{15}N parameters are used) and BP-triplet chemical shifts for ^{15}N (upper panel) and $^{1}H^{N}$ (lower panel). (b) Imino chemical shift map based on calculated RC shifts. The calculated imino resonance of each BP-triplet is shown as a marker on the map. (c) Enlarged view of Imino chemical shift map.

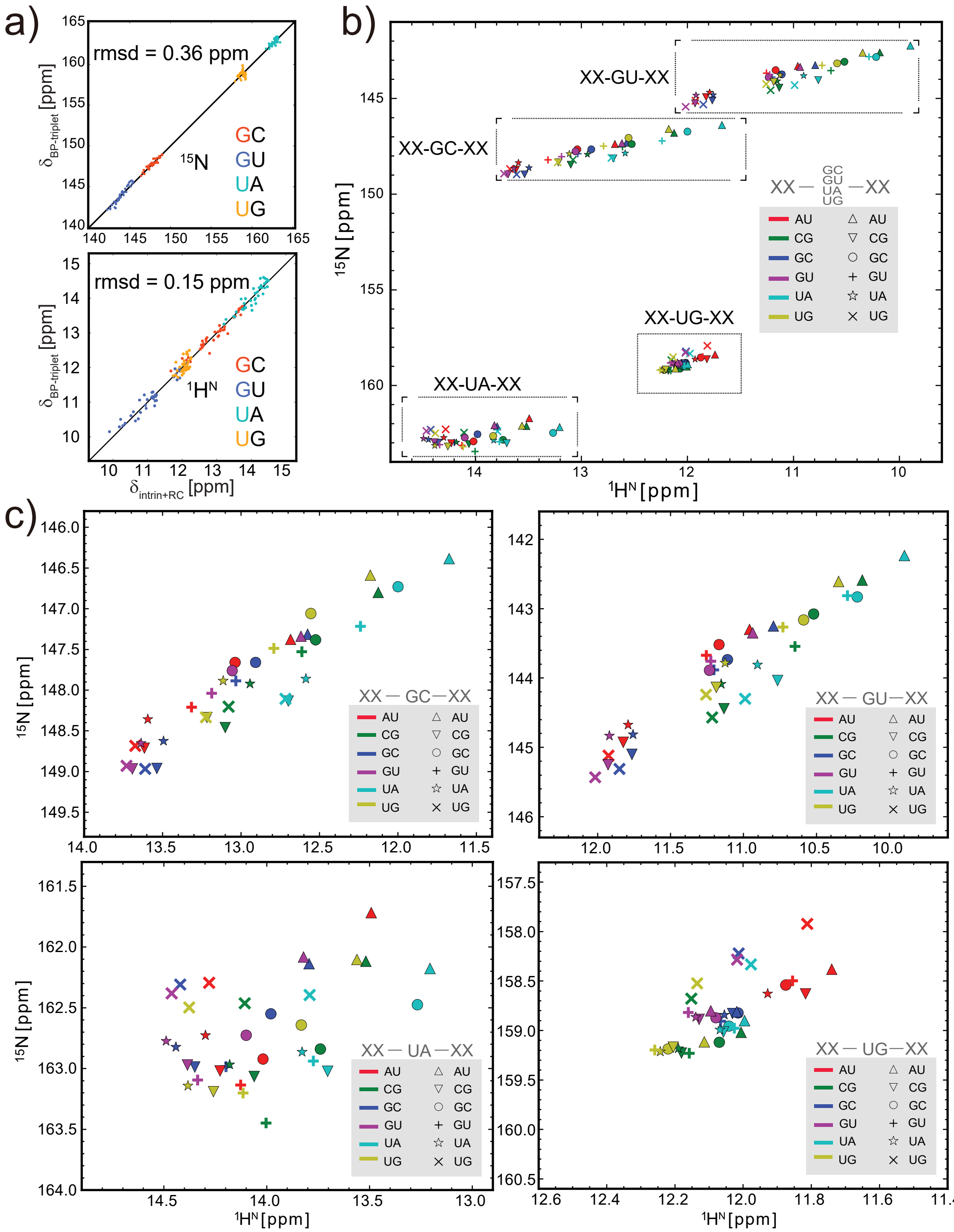

Figure S11. Comparison of ^{15}N (upper panel) and $^{1}H^{N}$ (lower panel) chemical shifts for the guanine in the CG closing base pair of UUCG tetraloops. The secondary structures of UUCG motifs are shown on top of each panel and the guanine of interest is highlighted in red. The horizontal line in each subplot marks the sum of chemical shift contributions from the central CG base pair and the base pair immediately below it. Resonances colored in cyan and black represent the total chemical shifts of the highlighted guanine by including the contribution from UUCG tetraloop through two approaches: prediction using the BP-triplet table (cyan); RC shift calculation using the original parameter set (black). The resonances colored in red represent experimental values.

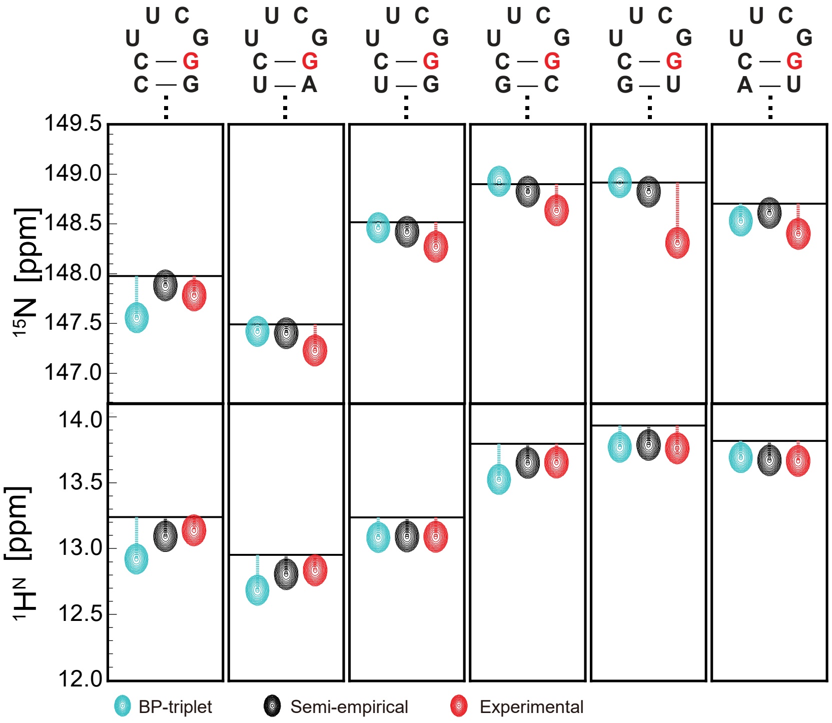

Figure S12. On-resonance $R_{1\rho}$ profiles of residues G164, U167, G174, G175 and G176 in P5c stem of P5abc. All the data were jointly fit to a simple two-state model. The error bars represent standard deviations (s.d.) estimated using Mente Carlo simulation with 50 iterations.

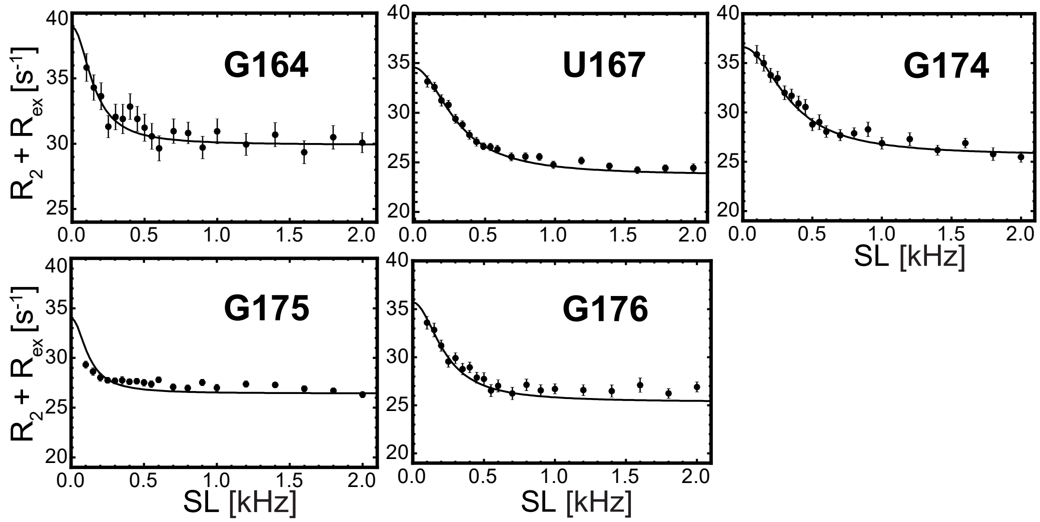

Figure S13. ^{15}N CEST profiles of residues G164, U167, G174, G175 and G176 in P5c stem of P5abc.

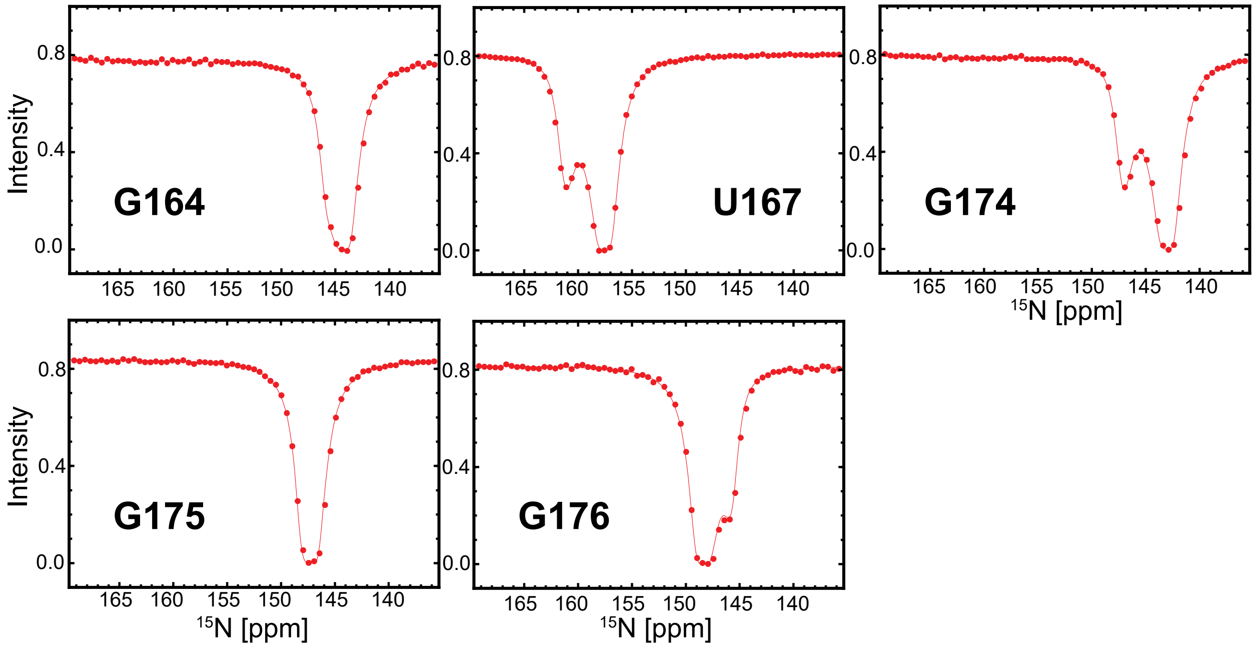

Figure S14. A hairpin sample designed to mimic the GG BP-triplet in P5abc excited state (ES). (a) Secondary structure of GG1 hairpin designed to produce BP-triplets of G176 and G164 in P5abc^{ES}. Residues 5-7 and 24-26 in GG1 correspond to residues 164-166 and 174-176 in P5abc^{ES} (see Fig. 7a), respectively. The G·G mismatch mimicking G164 and G176 of P5abc^{ES} are indicated in the dashed box. (b) A double-stranded RNA construct designed to distinguish one guanine resonance in G·G mismatch from the other, in which the stem of GG1 is concatenated to a stretch of AU base pairs to ensure enough length for efficient *in vitro* transcription. In this construct, G5 and G26 are used to mimic G164 and G176, respectively. Based on this construct, two samples were prepared with either forward strand (red) or reserve strand (green) isotope-labeled. (c) SOFAST ^{1}H-^{15}N HMQC spectrum of unlabeled GG1 hairpin (gray) overlaid with ^{1}H-^{15}N HSQC spectra of two strand-specifically labeled RNAs (red and green). Resonances from the GG1 hairpin are assigned in the figure. G5 and G26 from G·G mismatch are indicated by labels in bold font. Two unassigned resonances between 10 ppm and 11 ppm (orange arrows) are likely due to alternative local structure around G·G mismatch, which were also observed in other hairpin samples with similar G·G mismatch.

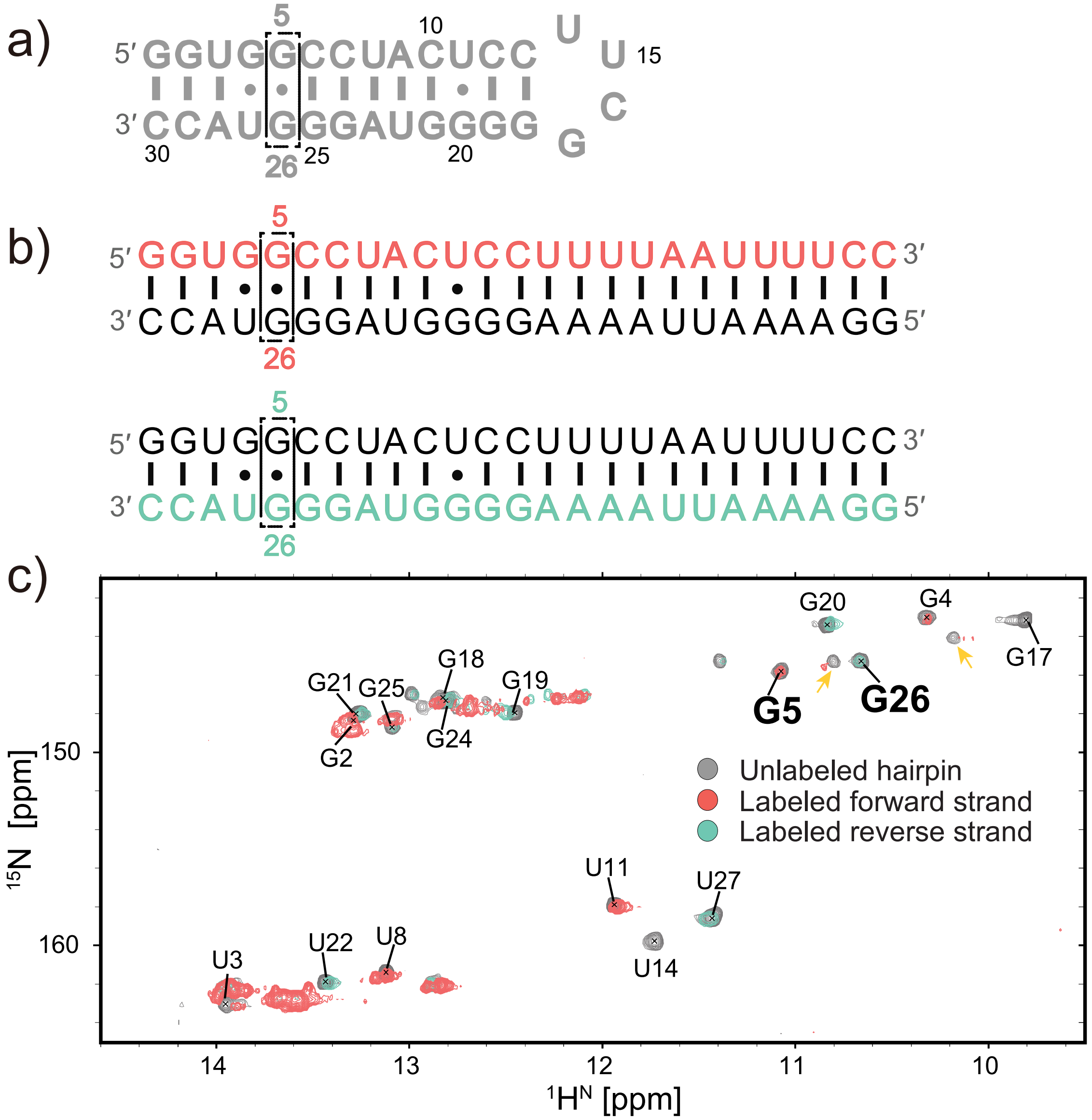

Figure S15. 2D ^{1}H-^{15}N HSQC spectrum (resonances colored in grey and red) of an unlabeled hairpin sample with pentaloop resembling P5c loop. This hairpin sample was prepared to collect chemical shifts of the BP-triplet centered on residue U167^{ES} of P5abc (corresponding to U11 of this hairpin RNA, shown as the red resonance). In addition, a simulated resonance (colored in blue) is placed on the spectrum to indicate the predicted chemical shifts of the BP-triplet CG-UA-UA. This BP-triplet, except that the opened U·A is replaced with a Watson-Crick UA base pair, is the same as the one centered on U11.

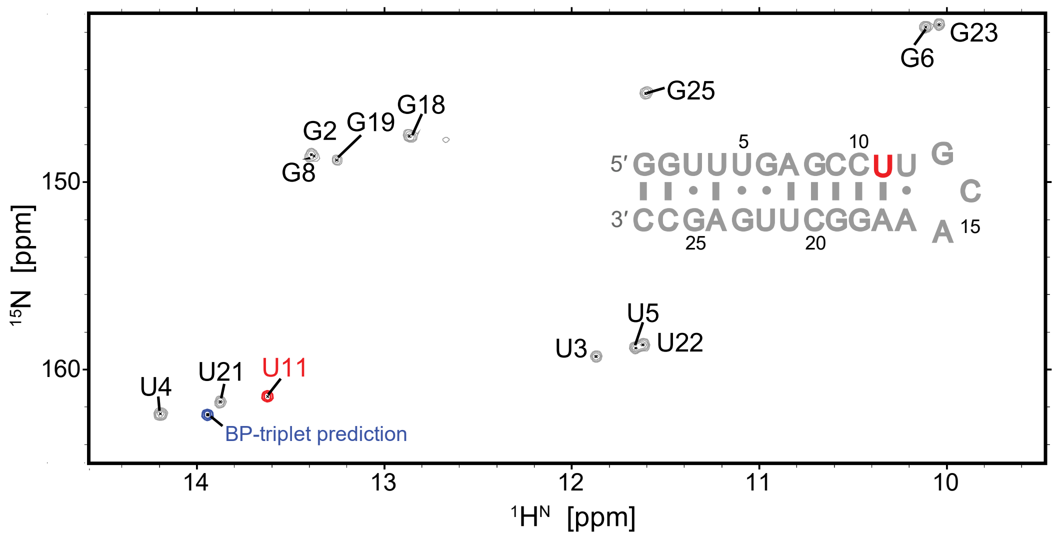

Supplementary Tables

Table S1. Hairpin RNA sequences and BMRB items in the training dataset.

RNA sample	Sequence or BMRB ID
HP15	GGAUUCGUGGCACUUCGGUGCUAUGAGUCC
HP16	GGGCAAUAUGUCCUUCGGGGUGUAUUGUCC
HP17	GGGUUGACUUUGCUUCGGUAGAGUUAACCC
HP18	GGUCGUGUUCUACUUCGGUAGGGCGCGACC
HP19	GGCCAUUGGAAUCUUCGGGUUUCGAUGGCC
HP20	GGUGUUAAGGAGCUUCGGCUCUUUAGUACC
HP21	GGGGGGAUGUUGCUUCGGUGAUAUCCCUCC
HP22	GGAAAGAGCUCGCUUCGGCGGGUUCUUUCC
HP23-GC	GGACGCCUGCGACUUCGGUUGUAGGUGUCC
HP23-GU3	GGACGCCUGGGACUUCGGUUUUAGGUGUCC
HP24	GGACAGGUGCUGCUUCGGCGGCAUUUGUCC
HP25	GGGCUGACUGGACUUCGGUUUGGUCAGUCC
HP26	GGAUCAUCGGGGCUUCGGUCUUGAUGGUCC
HP27	GGGUGGUUCCGUCUUCGGACGGAACCGUCC
HP28	GGAACGAGGUGACUUCGGUCGUUUUGUUCC
Rest01	GGACUGUAAGUUCUUCGGAAUUUGUGGUCC
Rest02	GGUUAUUUCUUACUUCGGUGAGGGGUAACC
Rest03	GGAAUGAGUUAGCUUCGGCUGGUUUAUUCC
Rest04	GGUCUUGAAAUACUUCGGUAUUUUGGGGCC
Rest05	GGGGCUUACUAUCUUCGGAUAGUAGGUCCC
Rest06	GGGCAGAUCAUGCUUCGGCGUGGUUUGUCC
Rest07	GGGUGUCUUUUACUUCGGUGGAAGACAUCC
Rest08	GGUCAGUGGUCGCUUCGGCGGCCGCUGACC
Rest09	GGAUAUGCACCUCUUCGGGGGUGUGUGUCC
Rest10	GGCGUGUUGGGUCUUCGGACCCAAUGCGCC
HP-GU	GGAUUCGGAUUGGUUCGCUAGUCUGGAUCC
PL01	GGGCCUGCCGGAAACGUUCCGGUAGGCCC
PL02	GGGCUCGUCGGUAACGUACCGAUGAGCCC
PL03	GGGCUGUUCGGGAACGUUCCGAACGGCCC
PL04	GGGUUCGUCGGCAACGUGCCGACGAGCCC
BMRB ID	4125 4135 4175 4226 4250 4253 4750 4780 4816 5007
	5046 5256 5321 5371 5395 5528 5553 5614 5632 5655
	5705 5834 5852 5919 5932 5962 5980 6062 6076 6077
	6094 6115 6239 6320 6485 6543 6562 6633 6652 6756
	7098 7230 7403 7404 7405 10014 11014 15080 15319 15417
	15538 15571 15656 15697 15745 15780 15781 15786 15856 15858
	16479 16714 16950 16951 16952 16953 17188 17292 17309 17316
	17326 17406 17436 17520 17566 17567 17568 17671 17682 17860
	17861 17901 17941 17961 17972 18239 18240 18336 18503 18515
	18532 18534 18656 18838 18891 18892 18894 18975 19018 19039
	19040 19545 19634 19662 19692 25049 25163 25291 25415 25416
	25603 25604 25654 25780 25781 25784 25785 25811 25867 26032
	26033 26568 26842 26938 30026 30046 30049 30051 30108 30132
	30224 30257 30258 30268 30282 30283 34038 34100

Table S2. BP-triplet lookup table that relates each BP-triplet to the average experimental imino chemical shifts of multiple occurrences.

BP-triplet	Chemical Shift of ^{15}N [ppm]	Standard deviation ^{15}N [ppm]	Chemical Shift of $^{1}H^{N}$ [ppm]	Standard deviation $^{1}H^{N}$ [ppm]	Total occurrences of ^{15}N	Total occurrences of $^{1}H^{N}$	Occurrences of ^{15}N & $^{1}H^{N}$ in hairpin RNAs
AU-GC-AU	147.24	0.22	12.82	0.07	9	18	2
AU-GC-CG	148.57	0.22	13.61	0.08	20	27	3
AU-GC-GC	147.68	0.24	13.03	0.09	13	23	2
AU-GC-GU	148.40	0.28	12.98	0.06	2	2	2
AU-GC-UA	148.52	0.14	13.61	0.07	16	22	2
AU-GC-UG	148.62	0.19	13.71	0.02	3	3	2
AU-GU-AU	142.88	0.16	10.82	0.06	5	6	2
AU-GU-CG	145.53	0.28	11.81	0.07	4	6	2
AU-GU-GC	143.49	0.03	11.10	0.06	2	4	2
AU-GU-GU	143.40	0.03	11.05	0.02	2	2	2
AU-GU-UA	145.07	0.08	11.96	0.04	3	3	3
AU-GU-UG	145.04	0.01	11.70	0.07	2	2	2
AU-UA-AU	161.93	0.17	13.48	0.07	20	24	3
AU-UA-CG	162.76	0.18	14.31	0.08	13	31	4
AU-UA-GC	162.30	0.07	13.65	0.08	7	15	2
AU-UA-GU	162.81	0.21	13.85	0.06	4	5	2
AU-UA-UA	162.62	0.15	14.31	0.08	7	14	3
AU-UA-UG	162.35	0.13	14.37	0.05	4	5	2
AU-UG-AU	158.60	0.10	11.76	0.08	2	3	2
AU-UG-CG	158.87	0.18	12.16	0.08	7	8	4
AU-UG-GC	158.78	0.13	11.72	0.04	3	4	3
AU-UG-GU	158.87	0.07	11.91	0.09	3	3	3
AU-UG-UA	159.01	0.11	12.01	0.03	3	3	3
AU-UG-UG	157.91	0.06	11.78	0.05	2	2	2
CG-GC-AU	146.56	0.19	12.21	0.11	11	13	5
CG-GC-CG	147.92	0.11	13.06	0.07	15	19	3
CG-GC-GC	147.11	0.11	12.53	0.07	15	22	6
CG-GC-GU	147.76	0.17	12.63	0.14	4	5	2
CG-GC-UA	147.68	0.19	13.03	0.08	14	19	2
CG-GC-UG	147.99	0.23	13.22	0.09	6	8	2
CG-GU-AU	142.40	0.16	10.29	0.01	3	3	3
CG-GU-CG	144.76	0.12	11.16	0.06	6	6	3
CG-GU-GC	143.15	0.16	10.61	0.07	9	9	3
CG-GU-GU	143.27	0.32	10.46	0.06	3	4	2
CG-GU-UA	144.43	0.15	11.30	0.08	3	4	3
CG-GU-UG	144.36	0.06	11.04	0.01	2	2	2
CG-UA-AU	161.92	0.30	13.46	0.06	5	9	2
CG-UA-CG	162.98	0.13	14.28	0.07	20	34	2
CG-UA-GC	162.35	0.23	13.69	0.07	13	23	2
CG-UA-GU	162.70	0.14	13.79	0.04	4	4	3
CG-UA-UA	162.67	0.22	14.19	0.05	12	19	2
CG-UA-UG	162.22	0.06	14.15	0.05	6	6	2
CG-UG-AU	158.61	0.06	11.90	0.05	2	3	2
CG-UG-CG	158.25	0.12	12.18	0.07	3	5	3
CG-UG-GC	158.71	0.13	11.96	0.12	8	8	3
CG-UG-GU	158.79	0.11	12.11	0.01	2	2	2
CG-UG-UA	158.15	0.16	11.96	0.06	2	4	2
CG-UG-UG	158.05	0.11	11.95	0.09	3	4	2
GC-GC-AU	147.58	0.18	12.58	0.11	28	43	5
GC-GC-CG	148.94	0.21	13.48	0.09	20	32	6
GC-GC-GC	147.92	0.14	12.79	0.10	11	17	2
GC-GC-GU	148.43	0.34	12.90	0.08	5	6	3
GC-GC-UA	148.74	0.21	13.45	0.07	12	27	6

GC-GC-UG	148.88	0.18	13.62	0.03	7	11	4
GC-GU-AU	143.15	0.15	10.70	0.05	6	8	2
GC-GU-CG	145.69	0.19	11.75	0.09	17	23	7
GC-GU-GC	143.61	0.30	11.13	0.08	5	7	3
GC-GU-GU	143.72	0.00	10.89	0.07	2	2	2
GC-GU-UA	145.26	0.21	11.73	0.04	7	8	4
GC-GU-UG	145.38	0.03	11.64	0.07	2	2	2
GC-UA-AU	162.27	0.09	13.72	0.04	14	18	3
GC-UA-CG	163.20	0.14	14.61	0.07	31	45	11
GC-UA-GC	162.50	0.10	13.94	0.08	12	23	2
GC-UA-GU	162.84	0.21	14.07	0.06	5	5	3
GC-UA-UA	162.89	0.11	14.55	0.08	20	30	5
GC-UA-UG	162.40	0.12	14.47	0.07	5	5	3
GC-UG-AU	158.90	0.29	11.92	0.12	5	5	3
GC-UG-CG	159.35	0.23	12.40	0.08	14	20	7
GC-UG-GC	159.26	0.21	11.93	0.06	6	6	3
GC-UG-GU	159.57	0.03	12.12	0.07	3	3	3
GC-UG-UA	159.34	0.15	12.19	0.11	4	6	2
GC-UG-UG	158.54	0.03	12.10	0.06	2	3	2
GU-GC-AU	147.42	0.16	12.68	0.05	2	5	2
GU-GC-CG	148.93	0.13	13.52	0.10	7	10	3
GU-GC-GC	147.56	0.20	12.92	0.11	6	7	2
GU-GC-GU	148.46	0.28	13.08	0.09	4	4	2
GU-GC-UA	148.53	0.15	13.69	0.09	2	2	2
GU-GC-UG	148.92	0.27	13.77	0.02	3	3	2
GU-GU-AU	143.15	0.08	10.91	0.04	2	2	2
GU-GU-CG	145.75	0.08	11.90	0.02	2	3	2
GU-GU-GC	143.72	0.05	11.17	0.07	3	4	2
GU-GU-GU	143.66	0.15	11.21	0.03	2	2	2
GU-GU-UA	145.23	0.15	11.95	0.08	2	2	2
GU-GU-UG	145.61	0.00	11.95	0.00	2	2	2
GU-UA-AU	162.56	0.00	13.74	0.05	2	2	2
GU-UA-CG	163.40	0.21	14.58	0.10	8	8	5
GU-UA-GC	163.10	0.23	13.96	0.09	6	7	3
GU-UA-GU	163.29	0.08	14.13	0.03	2	2	2
GU-UA-UA	163.21	0.26	14.51	0.06	5	6	3
GU-UA-UG	162.24	0.17	14.34	0.10	2	2	2
GU-UG-AU	159.12	0.02	11.92	0.10	2	2	2
GU-UG-CG	159.32	0.17	12.39	0.05	2	2	2
GU-UG-GC	159.89	0.16	12.01	0.00	2	2	2
GU-UG-GU	159.85	0.44	12.37	0.06	2	2	2
GU-UG-UA	159.32	0.30	12.03	0.06	2	2	2
GU-UG-UG	158.68	0.17	12.08	0.08	2	2	2
UA-GC-AU	146.33	0.18	11.90	0.06	6	17	2
UA-GC-CG	147.97	0.17	12.75	0.08	17	27	2
UA-GC-GC	147.04	0.23	12.26	0.08	8	15	2
UA-GC-GU	147.29	0.18	12.17	0.08	2	3	2
UA-GC-UA	147.73	0.19	12.77	0.06	8	12	2
UA-GC-UG	147.89	0.10	12.86	0.07	7	11	3
UA-GU-AU	142.18	0.18	10.15	0.04	6	6	3
UA-GU-CG	144.32	0.12	11.06	0.05	5	5	3
UA-GU-GC	142.83	0.04	10.50	0.03	2	3	2
UA-GU-GU	142.75	0.18	10.32	0.00	2	2	2
UA-GU-UA	144.05	0.10	11.21	0.02	2	3	2
UA-GU-UG	144.33	0.36	11.07	0.01	2	2	2
UA-UA-AU	162.25	0.12	13.26	0.07	3	4	2
UA-UA-CG	163.15	0.27	13.98	0.08	28	47	6
UA-UA-GC	162.64	0.12	13.39	0.07	5	9	2
UA-UA-GU	163.19	0.15	13.68	0.13	2	2	2
UA-UA-UA	163.08	0.20	13.99	0.12	8	11	2
UA-UA-UG	162.52	0.16	13.85	0.12	5	7	3
UA-UG-AU	158.54	0.17	11.78	0.09	6	6	3

UA-UG-CG	158.75	0.30	12.08	0.05	3	5	2
UA-UG-GC	158.93	0.16	11.84	0.03	4	4	3
UA-UG-GU	159.06	0.21	11.96	0.17	2	2	2
UA-UG-UA	158.65	0.09	12.00	0.09	5	6	2
UA-UG-UG	158.45	0.42	11.89	0.17	2	2	2
UG-GC-AU	146.74	0.20	12.29	0.07	4	4	4
UG-GC-CG	147.98	0.20	13.10	0.09	5	6	2
UG-GC-GC	147.47	0.31	12.65	0.01	3	3	2
UG-GC-GU	147.67	0.29	12.41	0.07	3	3	2
UG-GC-UA	147.94	0.25	13.12	0.07	5	6	3
UG-GC-UG	148.11	0.26	13.18	0.06	5	5	2
UG-GU-AU	142.26	0.28	10.39	0.08	2	2	2
UG-GU-CG	144.20	0.03	11.16	0.07	3	3	3
UG-GU-GC	142.47	0.14	10.72	0.02	2	2	2
UG-GU-GU	142.37	0.09	10.52	0.04	2	2	2
UG-GU-UA	143.93	0.12	11.28	0.02	3	3	3
UG-GU-UG	143.97	0.16	11.21	0.09	2	2	2
UG-UA-AU	162.28	0.05	13.61	0.02	4	4	2
UG-UA-CG	163.40	0.13	14.58	0.08	4	7	3
UG-UA-GC	162.57	0.30	13.73	0.09	3	4	2
UG-UA-GU	163.12	0.01	14.00	0.06	2	2	2
UG-UA-UA	162.74	0.22	14.31	0.12	2	3	2
UG-UA-UG	162.73	0.04	14.42	0.03	2	2	2
UG-UG-AU	158.41	0.18	12.03	0.02	2	2	2
UG-UG-CG	158.70	0.17	12.50	0.10	2	2	2
UG-UG-GC	158.68	0.20	12.02	0.09	3	4	2
UG-UG-GU	158.97	0.24	12.29	0.07	2	2	2
UG-UG-UA	158.42	0.02	12.25	0.09	2	2	2
UG-UG-UG	158.29	0.14	12.45	0.10	2	2	2
				Total	853	1198	381

Table S3. Hairpin RNA sequences and BMRB items in the testing dataset.

RNA sample	Sequence / ID
Rest11	GGCCUUCUGUUGCUUCGGUAGCGGAAGGCC
Rest12	GGGAUGGCUCGGCUUCGGCCGAGCUAUUCC
Rest13	GGUGCGUUUCGGCUUCGGUUGGAACGUACC
Rest14	GGAGCUCGCUACCUUCGGGUAGCGGGUUCC
Rest15	GGAGGUACAGGCCUUCGGGUUUGUGCUUCC
Rest16	GGAGGCGGUGUUCUUCGGGACACUGCCUCC
Rest17	GGGAUACAGUCCCUUCGGGGGUUGUGUCCC
Rest18	GGUGUCGCGAUGCUUCGGUAUUGCGAUACC
Rest19	GGGGGUGCCAUUCUUCGGAAUGGCGUCCCC
UU1	GGCGUGUGUCUGGUUCGCCUGAUAUGCGCC
P5abc	GGCAGUACCAAGUCGCGAAAGCGAUGGCCUUGCAAAGGGUAUGGUAAUAAGCUGCC
Gln-riboswitch	GGCGUUGGCCCAGUUUAUCUGGGUGGAAGUAAGGUCUUUGGCCUGAAGCAACGCG
HIV-1 TAR	GGCAGAUCUGAGCCUGGGAGCUCUCUGCC
Bacterial A-site	GGCGUCACACGCUUCGGCGUGAAGUCGCC
BMRB ID	4346 4694 15257 15859 25164* 30452 34259

* A guanine residue in this entry (G20) is pre-excluded because it is severely affected by the pseudoknot.

Table S4. The root-mean-square deviations (rmsd) between experimental chemical shifts in the testing dataset and chemical shifts predicted by BP-triplet lookup tables derived from the training dataset.

Training dataset	Testing dataset	GC/GU		UA/UG		All	
		N [ppm]	H [ppm]	N [ppm]	H [ppm]	N [ppm]	H [ppm]
TRAINING	TESTING	0.179	0.096	0.209	0.100	0.193	0.097
30 hairpins	BMRB	0.236	0.095	0.233	0.090	0.234	0.093
30 hairpins	TESTING	0.212	0.104	0.249	0.111	0.222	0.105
BMRB	TESTING	0.236	0.110	0.271	0.107	0.246	0.107

TRAINING: the original training dataset; **TESTING**: the original testing dataset**; 30 hairpins**: the data collected from the 30 hairpins in TRAINING; **BMRB**: BMRB entries in TRAINING.

Table S5. The root-mean-square deviations (rmsd) between experimental chemical shifts and the result calculated by ring-current model using BP-triplet (left column) or BP-pentalet (right column). BP-pentalet consists of the central base pair, two neighboring base pairs towards 5′, and two neighboring base pairs towards 3′. The A-form helix structures were built by RNAComposer.

Central base pair		rmsd (BP-triplet) [ppm]	rmsd (BP-pentalet) [ppm]
GC	N	0.266	0.262
	H	0.164	0.148
UA	N	0.348	0.341
	H	0.170	0.169
GU	N	0.613	0.609
	H	0.161	0.154
UG	N	0.599	0.601
	H	0.174	0.174

Table S6. The root-mean-square deviations (rmsd) between experimental chemical shifts and calculated chemical shifts for the guanine in the closing CG base pair of UUCG tetraloop. The contribution from the base pair immediately below CG and the intrinsic chemical shift are taken from Table 2. The RC shifts were calculated against three NMR structures (2KOC, 2M4Q and 5IEM) and two crystal structures (1F7Y and 5Y85) using different parameter sets: the superscript 'DC' means DC set; the superscript 'calib' means re-calibrated DC set with ^{15}N parameters optimized. For NMR structures, each conformation in the ensemble was calculated individually. For crystal structures, TL1 represents the first tetraloop while TL2 the second.

Structure		RC^{DC} H [ppm]	$RC^{DC}+EF^{DC}$ H [ppm]	RC^{DC} N [ppm]	RC^{calib} N [ppm]
2KOC (NMR)	1st	0.060	0.065	0.247	0.152
	2nd	0.062	0.085	0.239	0.147
	3rd	0.060	0.070	0.255	0.162
	4th	0.060	0.079	0.239	0.158
	5th	0.067	0.060	0.267	0.229
	6th	0.063	0.091	0.244	0.151
	7th	0.060	0.087	0.221	0.144
	8th	0.077	0.096	0.224	0.180
	9th	0.060	0.071	0.237	0.153
	10th	0.061	0.062	0.258	0.202
	11th	0.065	0.089	0.239	0.153
	12th	0.067	0.063	0.277	0.257
	13th	0.062	0.083	0.240	0.151
	14th	0.062	0.085	0.261	0.238
	15th	0.063	0.061	0.264	0.208
	16th	0.067	0.083	0.231	0.153
	17th	0.066	0.078	0.219	0.181
	18th	0.065	0.063	0.245	0.219
	19th	0.060	0.066	0.243	0.147
	20th	0.060	0.083	0.245	0.157
	Mean	0.060	0.074	0.244	0.158
2M4Q (NMR)	1st	0.144	0.068	0.356	0.478
	2nd	0.142	0.073	0.353	0.494
	3rd	0.142	0.121	0.352	0.515
	4th	0.147	0.119	0.323	0.388
	5th	0.155	0.092	0.357	0.530
	6th	0.143	0.120	0.347	0.535
	7th	0.155	0.080	0.371	0.575
	8th	0.071	0.120	0.302	0.251
	9th	0.119	0.084	0.332	0.377
	10th	0.147	0.144	0.355	0.561
	Mean	0.135	0.077	0.345	0.469
5IEM (NMR)	1st	0.098	0.063	0.285	0.409
	2nd	0.101	0.087	0.281	0.415
	3rd	0.091	0.064	0.268	0.392
	4th	0.105	0.079	0.291	0.435
	5th	0.101	0.066	0.284	0.408
	6th	0.082	0.060	0.250	0.363
	7th	0.096	0.074	0.275	0.421
	8th	0.096	0.063	0.283	0.402
	9th	0.079	0.074	0.286	0.351
	10th	0.097	0.070	0.282	0.427
	Mean	0.094	0.068	0.279	0.402
1F7Y (X-ray)	TL1	0.082	0.087	0.238	0.149
	TL2	0.060	0.071	0.271	0.247
5Y85 (X-ray)	TL1	0.061	0.060	0.272	0.274
	TL2	0.061	0.065	0.250	0.208

Table S7. Chemical shift changes due to single-nucleotide register shift in BP-triplets.

GS	ES	$\Delta\varpi_N$ [ppm]	$\Delta\varpi_H$ [ppm]	GS	ES	$\Delta\varpi_N$ [ppm]	$\Delta\varpi_H$ [ppm]
GU-GC-GC	GC-GC-GU	-0.87	0.03	GC-GC-GU	GU-GC-GC	0.87	-0.03
GC-GC-GU	GC-GC-GC	0.51	0.10	GC-GC-GC	GC-GC-GU	-0.51	-0.10
GU-GC-GC	GC-GC-GC	-0.36	0.13	GC-GC-GC	GU-GC-GC	0.36	-0.13
UA-UA-UA	UA-UA-UG	0.57	0.14	UA-UA-UG	UA-UA-UA	-0.57	-0.14
UG-UA-UA	UA-UA-UA	-0.34	0.31	UA-UA-UA	UG-UA-UA	0.34	-0.31
UG-UA-UA	UA-UA-UG	0.23	0.45	UA-UA-UG	UG-UA-UA	-0.23	-0.45
AU-GU-GC	AU-GU-GU	0.09	0.06	AU-GU-GU	AU-GU-GC	-0.09	-0.06
GU-GU-GU	GU-GU-GC	-0.06	0.04	GU-GU-GC	GU-GU-GU	0.06	-0.04
GU-GU-AU	GC-GU-AU	0.00	0.21	GC-GU-AU	GU-GU-AU	0.00	-0.21
GU-GU-GC	GC-GU-GU	0.00	0.27	GC-GU-GU	GU-GU-GC	0.00	-0.27
GU-GU-GU	GC-GU-GU	-0.06	0.32	GC-GU-GU	GU-GU-GU	0.06	-0.32
CG-UG-UA	CG-UG-UG	0.09	0.01	CG-UG-UG	CG-UG-UA	-0.09	-0.01
UG-UG-UG	UG-UG-UA	-0.13	0.20	UG-UG-UA	UG-UG-UG	0.13	-0.20
UG-UG-UA	UA-UG-UG	-0.04	0.36	UA-UG-UG	UG-UG-UA	0.04	-0.36
UG-UG-CG	UA-UG-CG	-0.05	0.42	UA-UG-CG	UG-UG-CG	0.05	-0.42
UG-UG-UG	UA-UG-UG	-0.17	0.56	UA-UG-UG	UG-UG-UG	0.17	-0.56
UG-UA-UG	UA-UG-UG	4.28	2.54	UA-UG-UG	UG-UA-UG	-4.28	-2.54
UG-UA-CG	UA-UG-CG	4.64	2.50	UA-UG-CG	UG-UA-CG	-4.64	-2.50
UG-UA-UG	UA-UG-UA	4.09	2.43	UA-UG-UA	UG-UA-UG	-4.09	-2.43
UG-UA-UA	UA-UG-UA	4.09	2.31	UA-UG-UA	UG-UA-UA	-4.09	-2.31
CG-UA-UA	CG-UG-UA	4.53	2.22	CG-UG-UA	CG-UA-UA	-4.53	-2.22
GU-GC-GU	GC-GU-GU	4.74	2.19	GC-GU-GU	GU-GC-GU	-4.74	-2.19
CG-UA-UG	CG-UG-UA	4.07	2.19	CG-UG-UA	CG-UA-UG	-4.07	-2.19
UG-UA-UG	UG-UG-UA	4.32	2.17	UG-UG-UA	UG-UA-UG	-4.32	-2.17
UG-UA-UA	UG-UG-UA	4.33	2.05	UG-UG-UA	UG-UA-UA	-4.33	-2.05
GC-GC-GU	GC-GU-GU	4.72	2.00	GC-GU-GU	GC-GC-GU	-4.72	-2.00
GU-GC-AU	GC-GU-AU	4.27	1.98	GC-GU-AU	GU-GC-AU	-4.27	-1.98
UA-UA-UG	UA-UG-UG	4.06	1.97	UA-UG-UG	UA-UA-UG	-4.06	-1.97
GU-GC-GU	GC-GU-GC	4.85	1.96	GC-GU-GC	GU-GC-GU	-4.85	-1.96
AU-GC-GC	AU-GU-GC	4.19	1.93	AU-GU-GC	AU-GC-GC	-4.19	-1.93
GU-GC-GU	GU-GU-GC	4.74	1.92	GU-GU-GC	GU-GC-GU	-4.74	-1.92
UA-UA-CG	UA-UG-CG	4.40	1.90	UA-UG-CG	UA-UA-CG	-4.40	-1.90
AU-GC-GU	AU-GU-GC	4.91	1.88	AU-GU-GC	AU-GC-GU	-4.91	-1.88
GC-GC-AU	GC-GU-AU	4.44	1.87	GC-GU-AU	GC-GC-AU	-4.44	-1.87
UA-UA-UG	UA-UG-UA	3.87	1.85	UA-UG-UA	UA-UA-UG	-3.87	-1.85
GU-GC-GC	GC-GU-GC	3.95	1.80	GC-GU-GC	GU-GC-GC	-3.95	-1.80
GC-GC-GU	GC-GU-GC	4.82	1.77	GC-GU-GC	GC-GC-GU	-4.82	-1.77
GU-GC-GC	GU-GU-GC	3.84	1.76	GU-GU-GC	GU-GC-GC	-3.84	-1.76

Table S8. The exchange parameters of P5abc obtained by fitting ^{15}N $R_{1\rho}$ data and $^{1}H^{N}$ CEST data to a two-state model (see *Methods*), including the experimental and predicted imino chemical shifts of ES.

Residue	R_2 (s^{-1})	k_{ex}	p_B	$\bar{\omega}_{GS}$ [ppm]	$\Delta\bar{\omega}$ [ppm]	$\bar{\omega}_{ES}^{exptl}$ [ppm]	$\bar{\omega}_{ES}^{pred}$ [ppm]
G174-N1	25.59±0.17	437±17 s^{-1}	2.6±0.1%	143.57	4.28±0.05	147.85	147.68
G174-H1	29.99±2.96			11.03	2.13±0.01	13.16	13.03
U167-N3	23.65±0.12			158.12	3.83±0.02	161.96	161.73
U167-H3	23.24±1.84			11.92	1.94±0.01	13.86	13.89
G176-N1	25.36±0.17			148.97	-2.70±0.04	146.27	145.60
G176-H1	48.19±7.29			13.57	-2.65±0.01	10.92	10.95
G164-N1	29.91±0.25			144.80	1.69±0.06	146.50	146.13
G164-H1	12.04±9.84			11.13	-0.05±0.09	11.08	11.37
G175-N1	25.95±0.21			147.76	1.23±0.06	148.99	149.03
G175-H1	54.41±11.21			13.05	0.23±0.01	13.28	13.39

Table S9. Spin lock power ($\omega_{SL}/2\pi$) and offset ($\Omega/2\pi$) used in ^{15}N $R_{1\rho}$ relaxation dispersion experiments of P5abc.

Residue	$\omega_{SL}/2\pi$ [Hz]	$\Omega/2\pi$ [Hz]
G174-N1<br>G175-N1<br>G164-N1	100	500,400,350,300,250,200,175,±150,125,100,±75,50,±25
	150	500,400,350,300,250,200,175,±150,125,100,±75,50,±25
	200	500,400,350,300,250,200,175,±150,125,100,±75,50,±25
	300	700,±500,400,350,±300,250,200,175,150,125,±100,±50
G176-N1	100	500,±400,±300,±200,±100, ±50,-125,-150,-250,-275,-350
	150	±500,±400,±300,±200,±100,±50,-150,-250,-350,-450
	200	±600,±400,±300,±200,±100,±50,-150,-250,-350,-450
	300	±800,±600,±400,±200,±100,±50,-150,-250,-300,-500
U167-N3	100	500,400,350,325,300,250,200,175,150,125,±100,75,±50,±25
	150	500,±400,350,300,250,±200,175,150,125,±100,75,±50
	200	500,±400,350,300,250,±200,175,150,125,±100,75,±50,
	300	700,±500,400,350,±300,250,200,175,150,125,±100,±50
