# Supplementary material for: Chemical shift prediction of RNA imino groups: application toward characterizing RNA excited states
Source: Nat Commun. 2021 Mar 11;12:1595. doi: 10.1038/s41467-021-21840-x (PMC7952389; doi:10.1038/s41467-021-21840-x)
Supplement: Supplementary file 3 — Reporting Summary [file 41467_2021_21840_MOESM3_ESM.pdf]

nature research

Corresponding author(s): Yi Xue

Last updated by author(s): Jan 29, 2021

Reporting Summary

Nature Research wishes to improve the reproducibility of the work that we publish. This form provides structure for consistency and transparency in reporting. For further information on Nature Research policies, see our Editorial Policies and the Editorial Policy Checklist.

Statistics

For all statistical analyses, confirm that the following items are present in the figure legend, table legend, main text, or Methods section.

n/a	Confirmed	
☐	☒	The exact sample size (*n*) for each experimental group/condition, given as a discrete number and unit of measurement
☐	☒	A statement on whether measurements were taken from distinct samples or whether the same sample was measured repeatedly
☒	☐	The statistical test(s) used AND whether they are one- or two-sided<br>*Only common tests should be described solely by name; describe more complex techniques in the Methods section.*
☒	☐	A description of all covariates tested
☒	☐	A description of any assumptions or corrections, such as tests of normality and adjustment for multiple comparisons
☐	☒	A full description of the statistical parameters including central tendency (e.g. means) or other basic estimates (e.g. regression coefficient) AND variation (e.g. standard deviation) or associated estimates of uncertainty (e.g. confidence intervals)
☒	☐	For null hypothesis testing, the test statistic (e.g. *F*, *t*, *r*) with confidence intervals, effect sizes, degrees of freedom and *P* value noted<br>*Give P values as exact values whenever suitable.*
☒	☐	For Bayesian analysis, information on the choice of priors and Markov chain Monte Carlo settings
☒	☐	For hierarchical and complex designs, identification of the appropriate level for tests and full reporting of outcomes
☐	☒	Estimates of effect sizes (e.g. Cohen's *d*, Pearson's *r*), indicating how they were calculated

Our web collection on statistics for biologists contains articles on many of the points above.

Software and code

Policy information about availability of computer code

Data collection	Bruker Topspin 3.2 (600MHz NMR spectrometer), Bruker Topspin 3.5 (800MHz NMR spectrometer)
Data analysis	NMRPipe 9.6, Sparky 3.113, ChemEx 0.6.1, custom python scripts (Python 2.7.12, numpy 1.11.0, matplotlib 1.5.1)

For manuscripts utilizing custom algorithms or software that are central to the research but not yet described in published literature, software must be made available to editors and reviewers. We strongly encourage code deposition in a community repository (e.g. GitHub). See the Nature Research guidelines for submitting code & software for further information.

Data

Policy information about availability of data

All manuscripts must include a data availability statement. This statement should provide the following information, where applicable:

- Accession codes, unique identifiers, or web links for publicly available datasets
- A list of figures that have associated raw data
- A description of any restrictions on data availability

All the key data are provided in the supporting tables, including accession codes of BMRB data that we analyzed, sequences of RNA constructs that we measured, chemical shift lookup table, and fitting results of NMR relaxation dispersion experiments. The NMR data acquired by us have been deposited in BMRB database with accession codes provided in the manuscript.

Field-specific reporting

Please select the one below that is the best fit for your research. If you are not sure, read the appropriate sections before making your selection.

☒ Life sciences ☐ Behavioural & social sciences ☐ Ecological, evolutionary & environmental sciences

For a reference copy of the document with all sections, see nature.com/documents/nr-reporting-summary-flat.pdf

Life sciences study design

All studies must disclose on these points even when the disclosure is negative.

Sample size	The number of RNA samples that we prepared were determined so that each base pair triplet appears at least twice. In this way the minimum requirement of statistics can be ensured for any base pair triplet, and at the same time potential mistakes such as mis-assignment can be recognized efficiently.
Data exclusions	No data were excluded from the analyses.
Replication	We constructed training dataset and testing dataset to avoid overfitting of data.
Randomization	The RNA constructs were allocated into experimental groups randomly. The training dataset and testing dataset were constructed randomly, too. We also used an additional scheme to design training/testing datasets to make sure the result is very much reproducible.
Blinding	We were blinded to group allocation during data collection and analysis.

Reporting for specific materials, systems and methods

We require information from authors about some types of materials, experimental systems and methods used in many studies. Here, indicate whether each material, system or method listed is relevant to your study. If you are not sure if a list item applies to your research, read the appropriate section before selecting a response.

Materials & experimental systems

n/a	Involved in the study
☒	☐ Antibodies
☒	☐ Eukaryotic cell lines
☒	☐ Palaeontology and archaeology
☒	☐ Animals and other organisms
☒	☐ Human research participants
☒	☐ Clinical data
☒	☐ Dual use research of concern

Methods

n/a	Involved in the study
☒	☐ ChIP-seq
☒	☐ Flow cytometry
☒	☐ MRI-based neuroimaging
